# System-Level Analysis of Transcriptional and Translational Regulatory Elements in *Streptomyces griseus*

**DOI:** 10.3389/fbioe.2022.844200

**Published:** 2022-02-25

**Authors:** Soonkyu Hwang, Namil Lee, Donghui Choe, Yongjae Lee, Woori Kim, Ji Hun Kim, Gahyeon Kim, Hyeseong Kim, Neung-Ho Ahn, Byoung-Hee Lee, Bernhard O. Palsson, Byung-Kwan Cho

**Affiliations:** ^1^ Department of Biological Sciences, Korea Advanced Institute of Science and Technology, Daejeon, South Korea; ^2^ KAIST Institute for the BioCentury, Korea Advanced Institute of Science and Technology, Daejeon, South Korea; ^3^ Biological and Genetic Resources Assessment Division, National Institute of Biological Resources, Incheon, South Korea; ^4^ Department of Bioengineering, University of California, San Diego, La Jolla, CA, United States; ^5^ Department of Pediatrics, University of California, San Diego, La Jolla, CA, United States; ^6^ Novo Nordisk Foundation Center for Biosustainability, Technical University of Denmark, Lyngby, Denmark

**Keywords:** *Streptomyces*, differential gene expression, regulatory elements, sigma factor, transcription factor, transcript 3′-end positions

## Abstract

Bacteria belonging to *Streptomyces* have the ability to produce a wide range of secondary metabolites through a shift from primary to secondary metabolism regulated by complex networks activated after vegetative growth terminates. Despite considerable effort to understand the regulatory elements governing gene expression related to primary and secondary metabolism in *Streptomyces*, system-level information remains limited. In this study, we integrated four multi-omics datasets from *Streptomyces griseus* NBRC 13350: RNA-seq, ribosome profiling, dRNA-seq, and Term-Seq, to analyze the regulatory elements of transcription and translation of differentially expressed genes during cell growth. With the functional enrichment of gene expression in different growth phases, one sigma factor regulon and four transcription factor regulons governing differential gene transcription patterns were found. In addition, the regulatory elements of transcription termination and post-transcriptional processing at transcript 3′-end positions were elucidated, including their conserved motifs, stem-loop RNA structures, and non-terminal locations within the polycistronic operons, and the potential regulatory elements of translation initiation and elongation such as 5′-UTR length, RNA structures at ribosome-bound sites, and codon usage were investigated. This comprehensive genetic information provides a foundational genetic resource for strain engineering to enhance secondary metabolite production in *Streptomyces*.

## Introduction

*Streptomyces* is a rich source of various secondary metabolites that exhibit a broad range of bioactivities, such as antimicrobial, antifungal, and anticancer (Worthen, 2008; Craney et al., 2013). These secondary metabolites are synthesized from precursor primary metabolites, such as acyl-CoA and amino acids, by a series of enzymatic reactions encoded in secondary metabolite biosynthetic gene clusters (smBGCs) in their genomes. Secondary metabolism is usually activated after vegetative growth terminates through a metabolic shift from primary to secondary metabolism, accompanied by morphological differentiation (Alam et al., 2010). This is because 1) both sporulation and secondary metabolism are needed to survive competition with other microorganisms under limited nutrient conditions (Hodgson, 2000; Van Wezel and Mcdowall, 2011), 2) the primary metabolites should be accumulated to obtain sufficient concentration as precursors of the secondary metabolites (Zabala et al., 2013), and 3) the production of secondary metabolites requires a lot of nutrients and energy, such as ATP and NAD(P)H, which can also be used for growth (Komatsu et al., 2010). Thus, to maintain the balance between cell growth and the production of secondary metabolites, *Streptomyces* should tightly regulate the metabolic shift during growth (Hodgson, 2000; Van Wezel and Mcdowall, 2011).

Dynamic gene expression during the metabolic shift is mostly controlled at the transcription initiation stage (Romero-Rodriguez et al., 2015). *Streptomyces* genomes encode approximately 60 sigma factors and hundreds of transcription factors that play critical roles in transcription initiation, thereby affecting the metabolic shift during growth (Romero-Rodriguez et al., 2015). For example, *S. griseus* NBRC 13350 encodes the A-factor cascade, which is a well-studied regulatory network that governs the metabolic shift (Ohnishi et al., 1999; Ohnishi et al., 2008). In addition, *Streptomyces* has post-transcriptional processing and translation regulatory elements to rapidly regulate the expression of smBGC-associated genes and developmental genes in response to dynamic cellular status (Jones, 2010). Several translational regulations have been reported in *Streptomyces*, such as ppGpp (Hesketh et al., 2007) and BldA., which is a unique tRNA for the rare codon TTA leucine (Higo et al., 2011; Hackl and Bechthold, 2015).

In this study, integrative analysis of four multi-omics datasets: RNA-seq, ribosome profiling, dRNA-seq, and Term-seq, was performed for *S. griseus* in three different growth phases. Functional enrichment of differentially expressed genes was investigated, and related regulatory elements were systematically analyzed for transcription initiation and termination, post-transcriptional processing, and translation initiation and elongation levels. We predicted one potential sigma factor regulon and four potential transcription factor regulons. Moreover, we also elucidated potential regulatory elements, such as sequence motif and stem-loop RNA structure at the 3′-end of transcripts. In particular, potential regulatory elements located in the middle of the polycistronic operon of hopanoid BGC and the potential cobalamin riboswitch element were investigated. Moreover, regulatory elements involved in translation initiation and elongation, in terms of 5′-UTR length, codon usage, and RNA structures, were studied. These systematic analyses of *S. griseus* provide a valuable genetic resource for understanding metabolic shifts during the growth phase.

## Materials and Methods

### Bacterial Cell Growth

*S. griseus* NBRC 13350 spores were inoculated in 50 ml R5-liquid complex medium (103 g/L sucrose, 0.25 g/L K_2_SO_4_, 10.12 g/L MgCl_2_∙6H_2_O, 10 g/L glucose, 0.1 g/L casamino acids, 5 g/L yeast extract, 5.73 g/L *N*-tris(hydroxymethyl)methyl-2-aminoethanesulfonic acid (pH 7.2), 0.08 mg/L ZnCl_2_, 0.4 mg/L FeCl_3_∙6H_2_O, 0.02 mg/L CuCl_2_∙2H_2_O, 0.02 mg/L MnCl_2_∙4H_2_O, 0.02 mg/L Na_2_B_4_O_7_∙10H_2_O, and 0.02 mg/L (NH_4_)_6_Mo_7_O_24_∙4H_2_O) and 8 g glass beads (3 ± 0.3 mm diameter) in a 250 ml baffled flask for the pre-culture. The preculture was grown for 24 h at 30 C with agitation at 250 rpm. The mycelium was then diluted 100-fold and transferred to fresh R5-medium for the main culture.

### Library Preparation and High-Throughput Sequencing for RNA-Seq and Ribosome Profiling

For RNA-seq and ribosome profiling, cells were sampled at exponential (E), transition (T), and stationary (S) phases. Additionally, for ribosome profiling samples, the ribosomes were stalled and cross-linked with mRNA by adding thiostrepton (Sigma) to cultures to attain a final concentration of 20 μM, as previously described (Kim et al., 2020). The cultures were incubated for 5 min at 30 C before harvesting. The protocol for library preparation for RNA-seq and ribosome profiling has been described previously (Kim et al., 2020). The constructed libraries were sequenced on the Illumina HiSeq 2,500 platform (Illumina Inc., San Diego, CA, United States) using a 100-bp single-end read recipe for RNA-seq, and a 50-bp single-end read recipe for ribosome profiling.

### Data Processing for RNA-Seq and Ribosome Profiling

Data processing for RNA-seq and ribosome profiling has been previously described (Kim et al., 2020). Briefly, sequencing results were de-multiplexed and processed using the CLC Genomics Workbench (Qiagen, Valencia, CA, United States). The trimmed reads were mapped to the reference genome (NCBI GenBank accession number: NC_010572). The mapped information was exported in BAM file format, and the number of mapped reads for each gene was the read count. Normalized RNA and ribosome protected footprint (RPF), fold changes, and principal component analysis (PCA) plots were generated using the DESeq2 package in R (Love et al., 2014). RNA, RPF, and translation efficiency (TE) values for each gene were normalized to transcripts per kilobase million (TPM).

### Library Preparation for dRNA-Seq and Term-Seq

Libraries for dRNA-seq and Term-seq were constructed as described previously (Lee et al., 2019; Hwang et al., 2021). The constructed dRNA-Seq and Term-Seq libraries were sequenced on the Illumina HiSeq 2,500 platform using the 100-bp single-end read recipe, and Illumina MiSeq using the 50-bp single-end read recipe.

### Data Processing for dRNA-Seq and Term-Seq

Transcription start sites (TSSs) and transcript 3′-end positions (TEPs) were determined as described previously with some modifications (Hwang et al., 2019; Lee et al., 2019; Hwang et al., 2021). The sequencing results were de-multiplexed and processed using the CLC Genomics Workbench. Raw sequencing reads were exported in FASTQ format and then mapped to the PhiX control sequences (NCBI GenBank accession number: NC_001422) to eliminate PhiX control reads with the following parameters: mismatch cost, 2; insertion cost, 3; deletion cost, 3; length fraction, 0.9; similarity fraction, 0.9; and non-specific matches, randomly mapped. The unmapped dRNA-seq and Term-seq reads were trimmed based on their overall quality (maximum ambiguous nucleotide score: 0.05) and length (minimum length: 15 nucleotides). The trimmed reads were mapped to the reference genome (NCBI GenBank accession number: NC_010572), ignoring the mapping of non-specific matches (mismatch cost, 2; insertion cost, 3; deletion cost, 3; length fraction, 0.9; and similarity cost, 0.9). After the mapping information was exported in a BAM file format, the 5′- and 3′-ends of mapped reads at each genomic position were counted as TSS and TEP peak raw counts, respectively.

For TSS determination, the peak with the maximum intensity among the peaks of a sub-cluster was selected. Peaks separated by less than 100 bp were clustered. Adjacent peaks with <10 standard deviations in their positions in the same cluster were further sub-clustered. As the 5′-end of the processed RNAs is expected to be abundantly located downstream of the TSS, peaks showing more than a 1.5-fold intensity at the position in TAP + than in TAP− conditions were selected. Finally, peaks that were not present in duplicates were removed. Assigned peaks were further manually curated according to RNA-seq profiles and peak enrichment patterns to determine the peak as the TSS.

For TEP determination, the enriched peak compared to adjacent peaks was selected using a machine learning algorithm with positive and negative control sets as inputs. For the positive control learning set, the peak with the maximum intensity among the peaks of a subcluster was selected to identify true-positive peaks among the peak shadows. Peaks separated by less than 100 bp were clustered. Adjacent peaks with <25 standard deviations of their positions in the same cluster were further subclustered. Low-intensity peaks with less than four counts were discarded to select enriched peaks. Peaks that were not present in either biological replicate were removed. The Z-score was an additional criterion for peak height enrichment (Lalanne et al., 2018). A high Z-score indicates that the peak height value is high compared to the total peak height distribution of adjacent peak shadows, reflecting peak height enrichment. Under the strict criterion of Z-score > 6, peaks with high values compared to adjacent peaks were selected. Peaks showing decreased RNA profiles near the TEPs were manually selected as positive control peaks. Negative control peaks were manually selected from −20 to +20 bp positions relative to the positive control peak positions. The TEPs were then searched using an in-house Python script based on the scikit-learn package. The Python script and KNN machine classifiers (pickled Python objects) are available at http://cholab.or.kr.

### Determination of Transcription Units and Transcription Unit Clusters

Transcription units (TUs) were determined by connecting TSS and TEP using a continuous RNA-seq profile. First, TSSs and TEPs were classified into two groups: coding and non-coding TUs. All possible combinations of TSS and TEP were tested for their association by calculating the normalized average RNA read count between 100 nt downstream region of the TSS and 100 nt upstream region of the TEP. If the distance between TSS and TEP was shorter than 200 nt, the normalized average RNA read count between TSS and TEP without the end criteria was calculated. The normalized average RNA read count of the 200 nt window region within the entire connected region was calculated and repeated for each +1 nt shifted window 200 nt upstream of the TEP. The connected region between TSS and TEP was classified as a TU when the average RNA read count of all windows was higher than 5% of the average RNA read count of the entire connected region for at least one growth phase. If the average raw RNA read count of the window was <5, it was excluded. Transcription unit clusters (TUCs) were defined as a group of connected TUs overlapping the TU region within at least 1 nt.

### Functional Enrichment Analysis

GO annotations of the total genes were exported from the UniProt proteome data (UniProt Proteome ID: UP000001685). GO_Biological_Process enrichment for each gene group was analyzed by a hypergeometric statistical test with Benjamini and Hochberg False Discovery Rate (FDR) correction using BiNGO (Maere et al., 2005). Enriched functions with FDR <0.05, high -log_10_(*p*-value), and not duplicated to other functions were selectively represented. A total of 43 smBGCs were determined by antiSMASH 5.0 (Blin et al., 2019).

### Read Density and Read-Through Fraction Analysis

To normalize the RNA read density, the maximum RNA read count between the -300 and +300 nt positions from TSS or TEP was normalized to 1, and RNA read counts at other positions were normalized relative to the maximum position. All RNA read counts for TSSs or TEPs at each position were then added and divided by the number of TSSs or TEPs. To determine the read-through fraction, the sum of normalized RNA counts at positions 0 to +300 nt was divided by the sum of normalized RNA read counts at positions −300 to 0 nt from the TEP. To calculate the RNA ratio of premature TUs to coding sequences (CDSs), the sum of normalized RNA read counts within each region was used.

### Motif Enrichment Analysis

The -10 motif of the promoter was searched by MEME (oops, *p* < 0.05) (Bailey et al., 2009) among the extracted sequences from −25 to +1 nt relative to the position of the TSS. The −35 motif of the promoter was searched by MEME (oops, *p* < 0.05) among the extracted sequences from -40 to -25 nt relative position of the TSS. The obtained motif sequences were aligned relative to the middle position of the conserved motif, and the 8 nt extracted sequence was used as the input for Weblogo 3 (Crooks et al., 2004). For consensus binding motifs of potential common regulons, including the sigma factor, the promoter list was selected by FIMO (input query: the extracted sequences from −40 to −25 nt relative position of sigma factor TSS; input database: the extracted sequences from −40 to −25 nt relative position of TSSs in each differential transcription pattern group, *p* < 0.01) (Grant et al., 2011), and MEME (oops, *p* < 0.05). The spacer length was calculated as the distance between the 3′-end of the −35 motif and the 5′-end of the −10 motif. Potential transcription factor binding motifs were searched by MEME (zoops) among the extracted sequences from −200 to +50 nt relative to the position of TSSs. The conserved TEP motif was searched using MEME (Zoops) among the extracted sequences from −40 to +20 nt relative to the position of TEP.

### RNA Structure Analysis

The fold free energy of the sequence from −40 to +1 nt relative to the position of TEP was calculated using RNAfold software (Lorenz et al., 2011). The interaction frequency between the two bases in the 100 nt upstream sequences was calculated based on the RNAfold results. For example, if the interaction between the −20 nt position and −18 nt position from the TSS was predicted in five out of 10 TEPs, the interaction frequency between the −20 nt position and −18 nt position was 0.5.

### Ribosome Pausing Analysis

The ribosome pausing score of each base position within the CDS was calculated by the 3′-end position counts of RPF reads accumulated at the position shifted by 14 nt downstream from the base position, then dividing by the average 3′-end position counts of RPF reads within the CDS. The first and the las 10 codons in the CDS were excluded from the calculation to remove the effects on translation initiation and termination. In addition, only genes with more than one RPF count per nucleotide were included in the calculation to remove the high pausing score calculated using the low average count of CDS. The ribosome pausing score for each A site codon was the sum of the pausing scores at the −1, 0, and +1 positions of the P site codon. The relative frequency of the codon of the top 1,000 pausing sites was calculated by subtracting the ratio of specific codons from the total codons from the ratio of codons among the top 1,000 pausing sites.

## Results

### Genome-Scale Determination of Gene Expression at Transcription and Translation Levels During Growth

**FIGURE 1:**
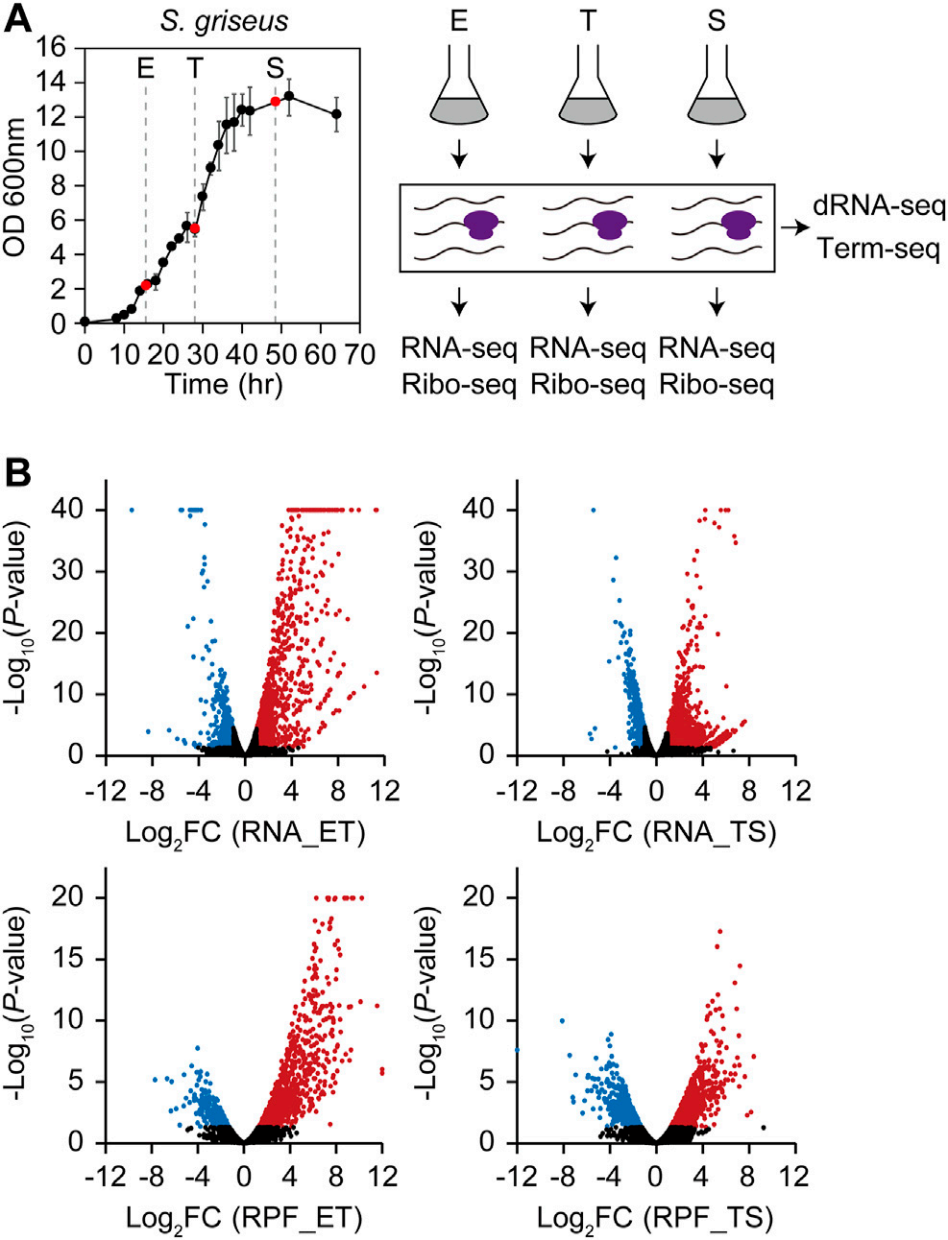
Four types of multi-omics data (RNA-seq, ribosome profiling, dRNA-seq, and Term-seq) of S. griseus in three different growth phases. **(A)** Growth profiles and overall experimental scheme for *S. griseus* multi-omics data generation. Liquid cell cultures were collected in duplicate at three growth phases, which are 15.5 h (E, early-exponential), 28 h (T, transition), and 48.5 h (S, stationary). RNA-seq and ribosome profiling libraries were prepared from each growth phase sample respectively, while dRNA-seq and Term-seq libraries were prepared from the pooled samples of the three growth phases. **(B)** Overall gene expression profiles of RNA-Seq (upper) and ribosome profiling (lower) represented by fold change and *p*-value of DESeq2 normalized gene expression from E to T (ET) and T to S (TS). Red dots are upregulated genes (Log_2_FC > 1, *p* < 0.05), blue dots are downregulated genes (log_2_FC < –1, *p* < 0.05), and black dots are the rest of the genes that are not differentially expressed. The dots with the −log_10_ *p*-value over 40 for RNA-seq or over 20 for ribosome profiling were also indicated at the coordinates of 40 and 20, respectively.

During the growth of *S. griseus*, dynamic changes in gene expression were expected to precisely coordinate the metabolic shift from primary to secondary. To identify the differentially expressed genes at the transcription and translation levels, RNA-seq and ribosome profiling libraries were prepared from the cells collected at exponential (E), transition (T), and stationary (S) phases (Figure 1A). RNA-seq and ribosome profiling data showed distinct gene expression profiles across each sample (Supplementary Figure S1A,B). RNA-seq and ribosome profiling reads were uniquely mapped to 6,960 genes in the genome, except for the long terminal inverted repeats at both ends (Ohnishi et al., 2008). RNA and ribosome-protected fragment (RPF) read counts were normalized and their fold changes (FC) with *p-*values of 1) E to T phase transition (ET) and 2) T to S phase transition (TS) were calculated (Figure 1B and Supplementary Table S1). Using the criteria of |log_2_ (FC)| > 1 and *p* < 0.05, a total of 1,086 upregulated and 568 downregulated genes and 1,312 upregulated, and 737 downregulated genes were selected from the ET and TS phase transitions at the transcription level, respectively. Using the same criteria, a total of 1,071 upregulated and 386 downregulated genes and 909 upregulated, and 669 downregulated genes were selected from the ET and TS phase transitions at the translation level, respectively. Overall, a total of 3,095 genes (44.5%) at the transcription level and 2,527 genes (36.3%) at the translation level were identified as differentially expressed genes (DEGs).

Differentially transcribed and translated genes showed a higher proportion of upregulated genes than downregulated genes at both the ET and TS phase transitions. The genes involved in morphological differentiation and various secondary metabolic pathways contribute to this overall expression trend. A total of 43 smBGCs were determined, and the distribution of normalized RNA read counts of their genes was calculated, except for two isorenieratene BGCs at the terminal inverted repeat regions (Supplementary Table S2). Median RNA read counts of smBGCs were much lower than those of total genes in the E phase, but they were more similar to those of total genes in the T and S phases, reflecting a gradually upregulated trend of smBGC genes (Supplementary Figure S2).

### Functional Enrichment of Differentially Expressed Genes During Growth

**FIGURE 2:**
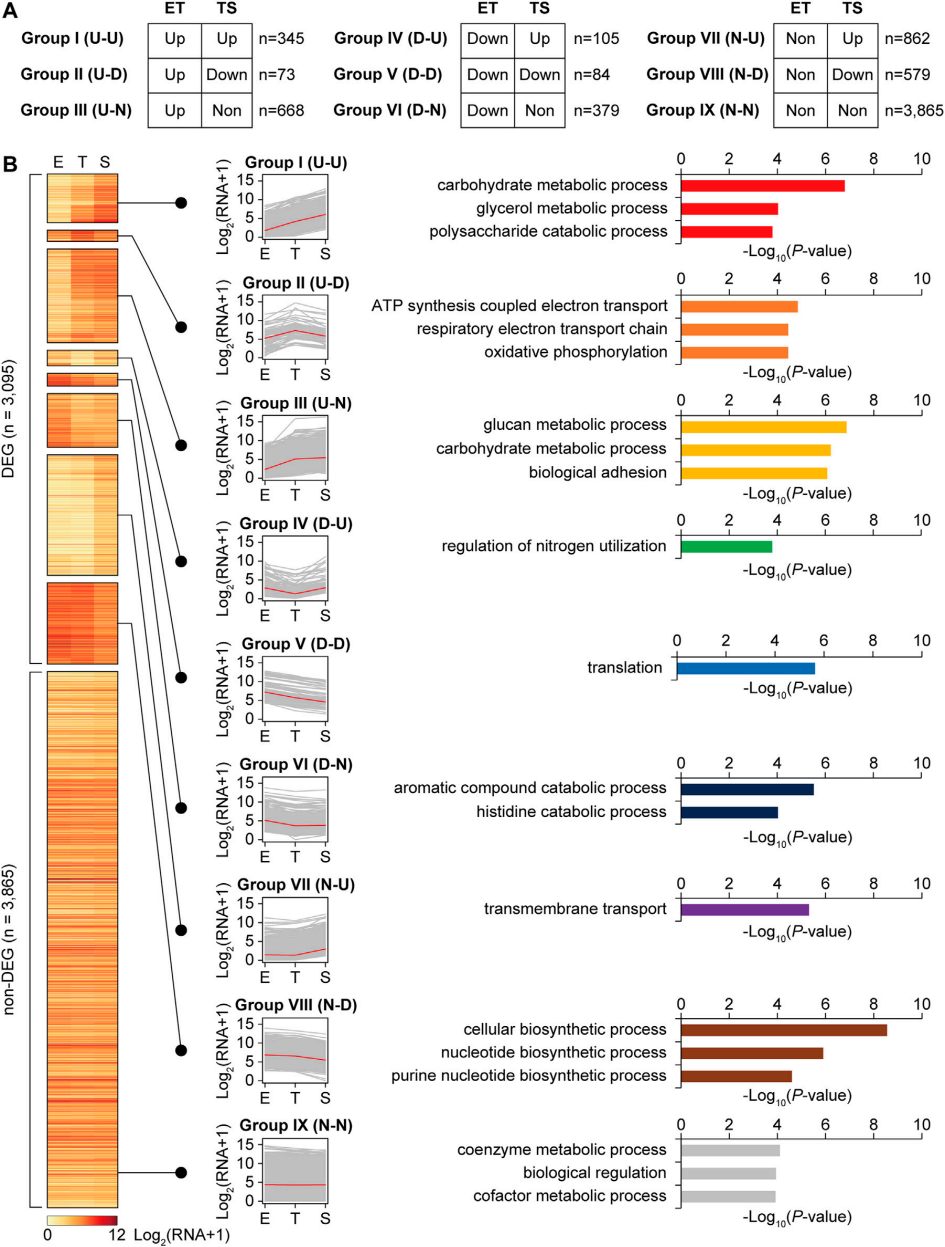
Clustering of total genes according to the transcription level patterns during growth, and enriched gene functions of each cluster group. **(A)** Total 6,960 genes were divided into differentially expressed genes (DEGs, |log_2_ fold change between E and T, or T and S phases| > 1, *p* < 0.05 by DESeq2), and non-DEGs (group IX). Further, DEGs were subdivided into eight transcription pattern groups according to their expression patterns. ‘ET’ and ‘TS’ indicate gene expressions at T phase compared to E phase, and at S phase compared to T phase, respectively. ‘U’, ‘D’, and ‘N’ indicate upregulated, down-regulated, and no significant change, respectively. **(B)** A heatmap of transcription level patterns of a total 6,960 genes, transcription pattern line graphs of each group, and the functional enrichment of genes in each group. Red line in the line graph is the median of transcription level among the genes. Selected enriched gene functions of each group were represented with their −log_10_ *p*-value (GO-term enrichment, GO_Biological_Processes, hypergeometric test, Benjamini and Hochberg False Discovery Rate (FDR) correction (*q* < 0.05)).

To analyze the transcription patterns according to the gene functions, total genes were categorized into nine expression groups (groups I-IX) based on their transcription patterns, which are a combination of upregulated (U), downregulated (D), or non-differential expression (N) for ET and TS phase transitions (Figure 2). The criteria of the upregulated (U) and downregulated (D) genes is described above (|log_2_ (FC)| > 1 and *p* < 0.05), and non-differential expression (N) genes include both the genes showing no significant difference in the transcription levels between the two phases, and the genes showing no significant expression. Functional enrichment of genes in each group was analyzed using the GO biological process. Overall, in the ET phase transition, the functions for cellular growth, including carbon source consumption, cell division by cell wall component recycling, and energy production, were upregulated (U). In contrast, the functions for translation, aromatic compound catabolism, and nitrogen utilization were downregulated (D). In the TS phase transition, the functions of carbon source consumption, nitrogen utilization, and transport were upregulated (U), whereas the functions in energy production, translation, and other biosynthetic processes were downregulated (D).

Individual smBGCs showed different expression patterns, even though they were categorized into the same BGC types (Supplementary Figure S3A). For example, melanin BGCs (BGC No.6, 20, and 36) showed different expression patterns, in which genes in one melanin BGC (No.6) belonged to either group I (U-U) or III (U-N), whereas several genes in the other two melanin BGCs (No.20 and 36) belonged to VIII (N-D) and VII (N-U), respectively (Supplementary Figure S3B). Melanin is a spore pigment that has broad biological functions in protecting cells from environmental stresses such as UV and oxidative stress (El-Naggar and El-Ewasy, 2017), and differential gene expression patterns between melanin BGCs suggest different functions of each melanin product. In terms of smBGC gene functional categories, biosynthetic genes were the most differentially expressed, whereas regulatory genes were less differentially expressed (Supplementary Figure S3C). Transport genes showed a high proportion of group VII (N-U) genes that may be activated after secondary metabolite biosynthesis to export them.

More than half of the streptomycin BGC genes were in group III (U-N), particularly for the genes involved in streptomycin biosynthetic reactions (Supplementary Figure S4). In contrast, pathway-specific activator gene *strR*, streptomycin transporter genes *strV* and *strW*, and other genes with unknown functions were mostly in group IX (N-N). This result was consistent with the A-factor regulatory cascade, in which *strR* is first induced to activate streptomycin biosynthetic genes (Horinouchi and Beppu, 1993). Although A-factor biosynthesis was predicted to be already activated in the E phase, we found genes *afsA* and *bprA* in groups VII (N-U) and IX (N-N), respectively. Other A-factor BGC genes also showed no change or upregulation during the T to S phase transition. In contrast, the A-factor responsive global regulator gene *adpA* was in group VIII (N-D); plausibly AdpA repressed its own transcription to maintain an appropriate level of AdpA to tightly regulate ordered differentiation and growth (Supplementary Figure S5). In summary, the functional enrichment of genes was differentially represented in different transcription pattern groups during growth to tightly regulate the dynamic metabolic shift, morphological differentiation, and secondary metabolism.

### Genome-Scale Determination of Transcription Start Sites and Transcript 3′-End Positions

Different regulatory elements can achieve differential gene expression at the transcriptional and translational levels. To elucidate the regulatory elements by determining genome-wide transcription start sites (TSSs) and transcript 3′-end positions (TEPs), dRNA-seq and Term-seq libraries were prepared from pooled samples of the three growth phases. A total of 1,887 TSSs were determined and subsequently classified into five categories based on their genomic positions relative to the adjacent genes (Supplementary Figure S6A and Supplementary Table S3). Furthermore, 2,115 TEPs were determined with subsequent selection of the enriched mapped peaks using machine learning and manual curation. They were categorized into six categories based on their genomic positions relative to adjacent genes (Supplementary Figure S6B and Supplementary Table S3). The normalized RNA read density in the −300 to +300 nt window from TSSs and TEPs showed apparent patterns of increase and decrease at the TSSs and TEPs, respectively (Supplementary Figure S6C,D). Moreover, the determined TSSs and TEPs were consistent with the mapping profiles of RNA-seq, confirming the integrity of the data (Supplementary Figure S6E).

### Sigma Factor Binding Motifs Regulating the Differential Gene Expressions

**FIGURE 3:**
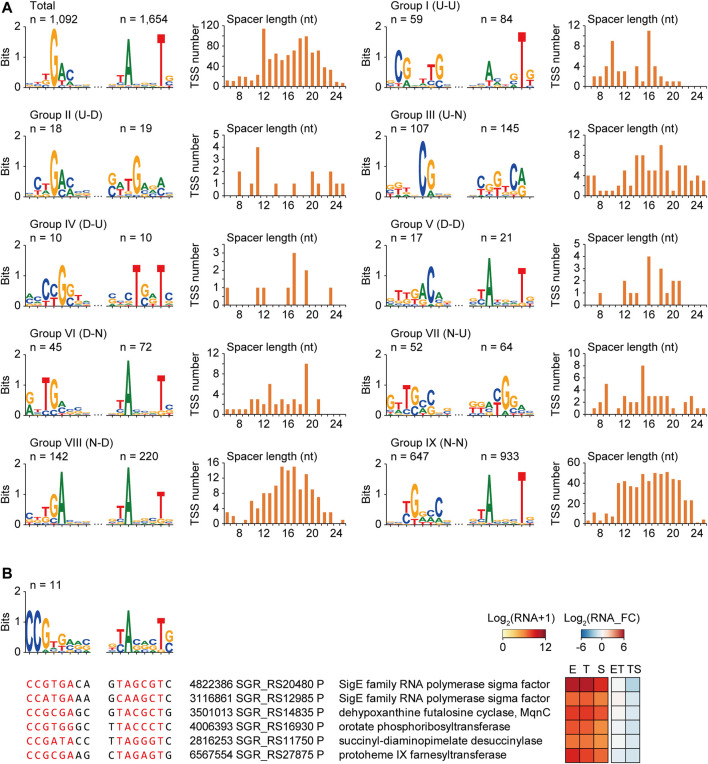
Potential consensus sigma factor binding motifs for different transcription pattern groups. **(A)** The −35 motifs, −10 motifs, and spacer length distributions of the promoters of total TSSs and different transcription pattern group TSSs. **(B)** An example of the consensus sigma factor-binding motif of group VIII (N–D) group, including respective sigma factor. Sequence alignments, gene functions, and RNA expressions of six selected promoters were represented. The six promoters were selected based on the motif similarity with the sigma factor SGR_RS20480 by FIMO and MEME with low *p*-value of −35 and −10 motif, and their gene functions.

Consensus −35 and −10 motif searches resulted in a total of 1,887 promoters, which were 5′-TGAC-3′ for the −35 motif and 5′-ANNNT-3′ for the −10 motif, respectively (Figure 3A). The -10 motif was well conserved in 87.7% of total TSSs, whereas the −35 motif was less conserved in 57.9% of total TSSs, respectively. Moreover, the spacer length distribution and the presence of other diverse lengths between −35 and −10 motifs were the most abundant at 12 nt, followed by 18 and 19 nt.

As diverse sigma factors are expected to be differentially expressed in response to cellular status, their binding to specific motifs would be different during growth, resulting in differential transcription patterns of downstream genes. To identify the sigma factor binding motifs governing specific differential transcription patterns, the conserved −35 and −10 motifs and the spacer length of the promoters of the nine transcription pattern groups in Figure 2 were searched (Figure 3A). The sigma factor-binding motifs were very diverse for the different groups, including the −10 motif group. This result suggests the involvement of a diverse set of sigma factors for each transcription group and even within the group. The genes having the similar binding motif with the motif of a certain sigma factor could be considered as either 1) potential regulon of the sigma factor binding to its own promoter for self-regulation, or 2) potential regulon of the other sigma factor binding to both the genes and the sigma factor (Chauhan et al., 2016; Hwang et al., 2019). Thus, all promoters in the same group were scanned to match the motifs of the sigma factor for the search of the potential regulon regulated by the identical sigma factor. TSSs were detected for 19 of the 54 sigma factors, and eight sigma factor genes were transcriptional DEGs [two for group III (U-N) and six for group VIII (N-D)]. The binding motif of the promoters was analyzed to identify potential sigma factor regulons affecting differential transcription patterns. Among them, only SGR_RS20480 had more than ten potential regulon genes, including functions in the cellular biosynthetic process (Figure 3B). Overall, a potential sigma factor regulon had a conserved sigma factor-binding motif, exhibiting similar expression patterns and gene functions during growth.

### Transcription Factor Binding Motifs Regulating the Differential Gene Expressions

**FIGURE 4:**
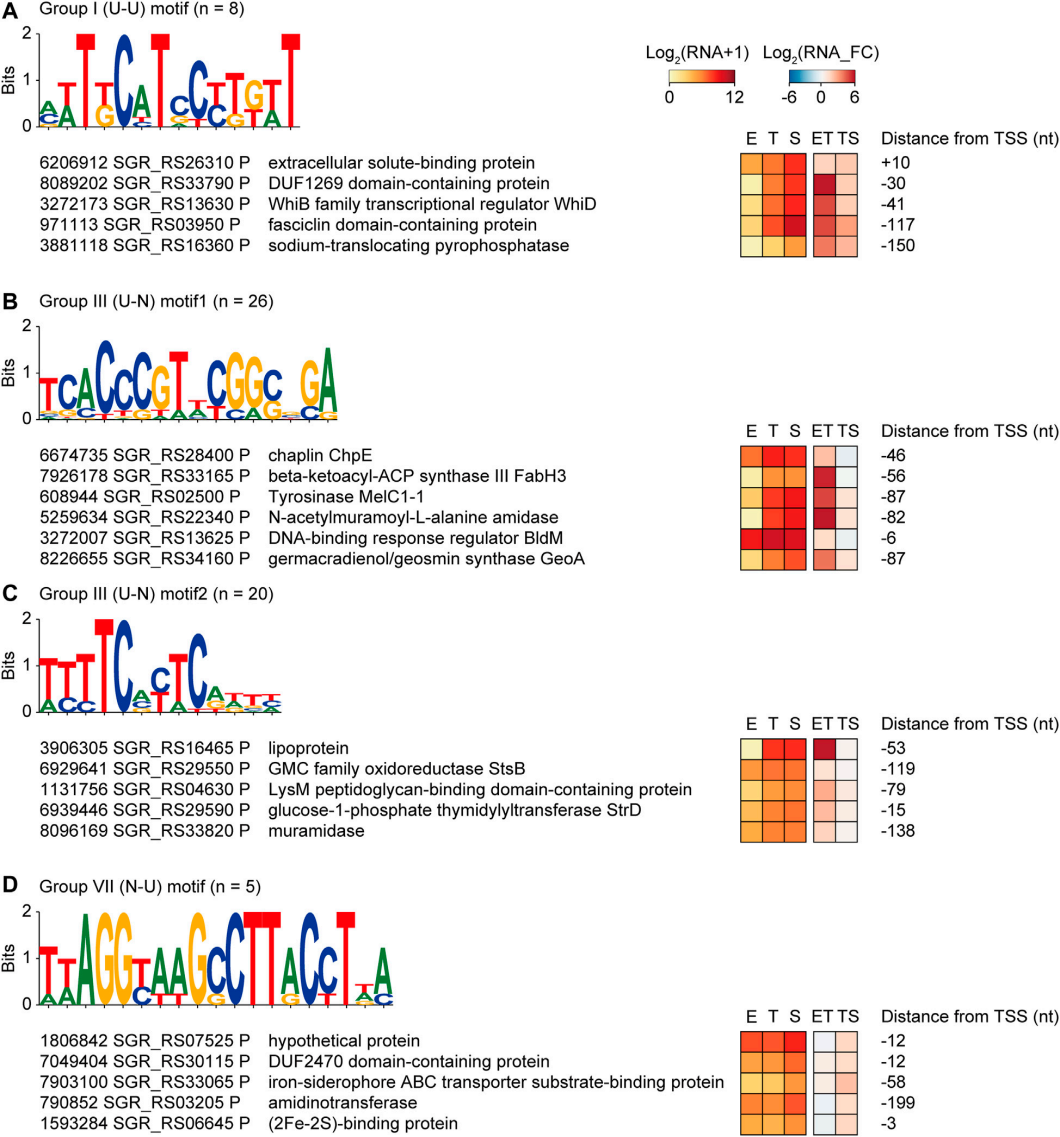
Four examples of potential transcription factor binding motifs found from genes of the same transcription pattern groups, which are **(A)** group I (U–U), **(B,C)** group III (U–N), and **(D)** group VII (N–U). Conserved motifs were detected by MEME with zoops parameter. The five or six promoters were selected based on the gene functions. RNA expression levels, fold changes, and distance from the TSS were also represented.

To identify the binding motifs of potential transcription factors and their regulons that have a common transcription pattern, conserved motifs in the −200 to +50 nt sequences of the TSSs of the same transcription pattern group were searched (zoop parameters for MEME). Four potential transcription factor binding motifs were predicted with their regulon genes: one in group I (U-U), two in group III (U-N), and one in group VII (N-U) (Figure 4). The transcription factors of these binding motifs could be 1) involved in the regulon and regulate their own transcription, or 2) not involved in the regulon but have an upper hierarchical position to the regulon genes in the transcription regulatory network.

First, a T-rich motif was found in group I (U-U) genes, including the WhiD transcription factor that governs sporulation (Figure 4A) (Jakimowicz et al., 2005). In addition, membrane and extracellular protein genes that are potentially involved in sporulation, such as DUF1269 domain-containing protein, extracellular solute-binding protein, fasciclin domain-containing protein, and sodium-translocating pyrophosphatase, were enriched in this regulon (Moody and Williamson, 2013).

Second, the motif 5′-TCACCCGTnCGGCnGA-3′ was found in group III (U-N), which was almost identical to the BldM dimeric binding motif (5′-TCACcCgnncGgGTGA-3′) (Figure 4B) (Al-Bassam et al., 2014). Indeed, BldM was also included in the 26 regulon genes, suggesting that the motif prediction method is valid. The regulon contained chaplin proteins for aerial mycelium formation as well as smBGC genes related to spore pigmentation, such as the tyrosinase gene in melanin BGC, beta-ketoacyl-ACP synthase II in HPQ melanin BGC, and a germacradienol/geosmin synthase GeoA gene in germacradienol/geosmin BGC. As BldM is known to play critical roles in aerial hyphae formation, it was confirmed that regulon genes have functional similarities with BldM.

Third, a CT-rich motif (5′-TTTTCnCTC-3′) was found in Group III (U-N) (Figure 4C). Genes involved in carbohydrate metabolic processes were found in the regulon, including the lipoprotein genes of griseobactin BGC and muramidase. Moreover, the StsB and StrD genes of the streptomycin biosynthesis pathway and LysM peptidoglycan-binding domain-containing protein in hopanoid BGC were present in this regulon, indicating that this transcription factor-binding motif is related to carbohydrate-type BGC genes.

Finally, the motif with 5′-TTA​GGT​AAG​CCT​TAC​CTT​A-3′ had an inverted repeat (Figure 4D). Five genes in group VII (N-U) contained this motif, including the heme iron utilization family DUF2470 domain-containing protein gene, iron-siderophore ABC transporter substrate-binding protein gene in HPQ melanin BGC, and (2Fe-2S)-binding protein gene. According to the enriched functions, specific iron-related transcription factors were predicted to bind to this motif. Indeed, this motif has a high sequence similarity to the binding motif of the iron-dependent transcriptional regulator DmdR1 in *Streptomyces* (Lee et al., 2020). Overall, four potential transcription factor regulons had a conserved transcription factor-binding motif, similar expression patterns during growth, and even similar gene functions.

### Regulatory Elements of Transcription Termination and Post-transcriptional Processing

**FIGURE 5:**
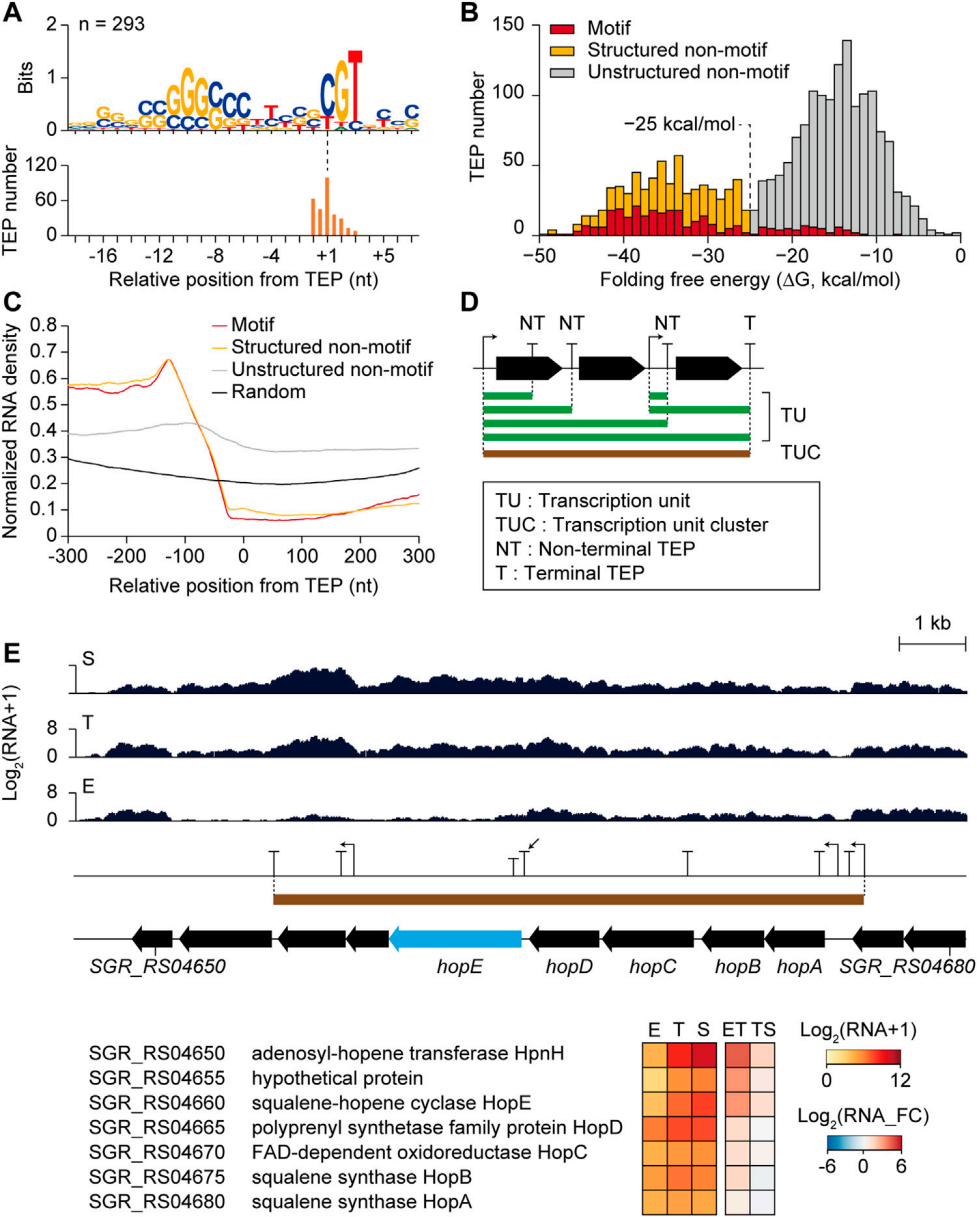
Potential transcription termination and post-transcriptional regulatory elements of transcription 3′-end positions (TEPs) affecting differential gene expressions. **(A)** Conserved TEP motifs searched by MEME with zoops parameter. **(B)** Folding free energy distribution of total TEPs (n = 2,115), and three subcategories which are Motif TEPs having the conserved motif, Structured non-motif TEPs with highly-structured RNA (<−25 kcal/mol) without the motif, and Unstructured non-motif TEPs with less-structured RNA (>−25 kcal/mol) without the motif. **(C)** Normalized RNA density of three TEP subcategories and random intergenic positions. **(D)** Schematic representation of transcription unit (TU), transcription unit cluster (TUC), non-terminal TEP (NT), and terminal TEP (T). **(E)** An RNA-seq profile example of the non-terminal TEP (indicated by arrow) having differentially expressed downstream gene (*hopE* gene with sky blue color). Gene functions, expression levels, and fold changes of the genes within the TUC were shown.

The differential transcription patterns of genes mainly result from transcriptional regulation by sigma factors and transcription factors at the transcription initiation level. However, the regulation of additional layers, including transcription termination and post-transcriptional processing, can affect transcription patterns. As TEPs are expected to contain these regulatory elements of transcription termination, and post-transcriptional processing, the sequence features of the TEP regions were investigated. The motif search of −40 to +20 nt sequences from 2,115 TEPs revealed a conserved motif in 293 TEPs (Figure 5A). These TEP with the motif (n = 293) had GC-rich stem at the -10 region and ‘CGT’ sequence with adjacent T-rich sequences at −5 to +5 nt region. These features were identical to those of *E. coli* intrinsic terminators, leading to rapid dissociation of the RNA polymerase elongation complex by destabilization of interactions in the RNA-DNA hybrid through RNA hairpin structure and pausing in the U-rich tract (Gusarov and Nudler, 1999). Considering the high GC content (>70%) of *Streptomyces* genome, a U-rich tract is rare across the genome; thus, it is likely to involve specific regulatory functions.

The distribution of folding free energy of the −40 to 0 nt sequences of the TEPs with the motif (**‘**Motif TEPs’ with the red color in Figure 5B) showed higher absolute energy compared to the TEPs without the motif, which showed a bimodal distribution with an intersect middle value of −25 kcal/mol (Figure 5B). As recent *in vivo* mapping of the 3′-end of Rho-dependent transcripts have revealed that stable RNA stem-loop structure was enriched that is protected from 3′ to 5′ exonuclease digestion, the RNA stem-loop structure was speculated as one of the most crucial regulatory elements both for transcription termination and degradation (Jones, 2010). Thus, the TEPs without the motif were further divided into ‘Structured non-motif TEPs’ (n = 441, yellow color in Figure 5B) with a higher absolute folding free energy than −25 kcal/mol and ‘Unstructured non-motif TEPs’ (n = 1,381, grey color in Figure 5B) with a lower absolute folding free energy than −25 kcal/mol. Abundant unstructured non-motif TEPs were presumably Rho-dependent termination sites with diffused termination patterns (Jin et al., 1992), or other distinct types of termination or RNA processing sites.

Alignment of −40 to +21 nt sequences of all three TEP subcategories revealed distinct features and random intergenic sequences (Supplementary Figure S7A). Although both Motif TEPs and Structured non-motif TEPs showed high absolute folding free energy of RNA structure, consensus GC stems at −30 and –10 nt regions were enriched more in Motif TEPs than in Structured non-motif TEPs. Moreover, Unstructured non-motif TEPs showed enriched ‘TG’ sequence at the TEP position, which was distinct to random intergenic sequences. The interaction frequency between each base at 100 nt upstream regions of the TEPs suggested that even Unstructured non-motif TEPs had weak but apparent RNA structures at the −30 and –10 nt regions compared to random intergenic sequences, confirming the presence of potential regulatory elements in Unstructured non-motif TEPs (Supplementary Figure S7B). These distinct sequence features were expected to include transcriptional regulatory elements that could affect transcript abundance changes in the TEP. Normalized RNA read density was dramatically decreased in the −100 to +1 nt region of Motif TEPs and Structured non-motif TEPs, and relatively less decreased in Unstructured non-motif TEPs, however, higher than in the random intergenic positions (Figure 5C).

### Regulatory Elements in Transcription Units

To analyze the differential changes in transcript abundance at the TEPs according to the growth phases, 1) the transcription units (TUs) connecting the TSS and the TEP having continuous RNA reads between them, 2) the transcription unit clusters (TUCs) involving all TU isoforms that have overlapping regions among themselves, 3) non-terminal TEPs located at the middle of each TUC, and 4) terminal TEPs located at the end of each TUC were defined (Figure 5D). A total of 2,093 TUs were determined and subsequently categorized as their included gene numbers and genomic positions, which were monocistronic, polycistronic, premature, antisense, and intergenic TUs (Supplementary Figure S8A and Supplementary Table S3). A total of 662 TUCs were determined, and the distribution of TU number among them gradually decreased as the TU number increased (Supplementary Figure S8B and Supplementary Table S3).

A total of 637 non-terminal TEPs without any downstream TSSs within TUC were selected to investigate the sole effect of non-terminal TEPs on the transcript abundance of the downstream gene. The non-terminal TEP of the structured non-motif group in the hopanoid BGC is shown as an example (Figure 5E). The RNA read profile of the downstream gene *hopE* was low in the E phase, but increased in the T and S phases. Although the upstream gene (*hopD*) and downstream gene (*hopE*) of the non-terminal TEP were both in group III (U-N), the fold change of *hopD* in the ET phase transition was 1.3, while that of *hopE* was 3.2, reflecting a sharp increase in *hopE* transcription. The *hopE* gene encodes a squalene-hopene cyclase, which is the key enzyme in hopanoid biosynthesis that cyclizes squalene to have ring structures, forming hopene (Siedenburg and Jendrossek, 2011; Ghimire et al., 2015). Upstream genes from the non-terminal TEPs were *hopA, hopB, hopC,* and *hopD*, governing the formation of squalene from isopentenyl diphosphate (IPP) and dimethylallyl diphosphate (DMAPP), whereas downstream genes (SGR_RS04655 and SGR_RS04650) from *hopE* seemed to be involved in the additional modification of adenosylhopene formation. Thus, *hopE* and downstream genes govern more hopene-specific reactions, whereas *hopA, hopB, hopC,* and *hopD* govern more general terpene biosynthetic reactions. Therefore, the non-terminal TEP between them was predicted to be a potential regulatory element of transcription termination or post-transcriptional processing for the efficient regulation of hopanoid BGC genes during growth.

In addition to the non-terminal TEP, 79 premature TUs could have regulatory elements affecting the differential transcription of the downstream gene. The RNA read count ratio of the premature TUs to the CDS indicated premature termination intensity, and their average and standard deviation at the three growth phases were diverse (Supplementary Figure S8C). Among them, the premature TU of the SGR_RS05925 gene was predicted to contain cobalamin riboswitch using the Rfam database (Griffiths-Jones et al., 2003; Borovok et al., 2006) and showed differential premature termination intensity during growth (Supplementary Figure S8D). In the E phase, the transcription initiation level at the TSS was sufficient, but premature termination at the premature TEP seemed to occur by cobalamin binding to the riboswitch, resulting in the repression of SGR_RS05925 transcription. In the T phase, the transcription of SGR_RS05925 was still low because of the lower transcription initiation level at the TSS compared to the E phase. However, in the S phase, the transcription of SGR_RS05925 increased due to the absence of premature termination, even though the transcription initiation level at the TSS was lower than in the E phase. In summary, transcriptional termination and post-transcriptional processing could affect differential gene expression through regulatory elements in TEPs.

### Regulatory Elements Governing Translation Initiation

**FIGURE 6:**
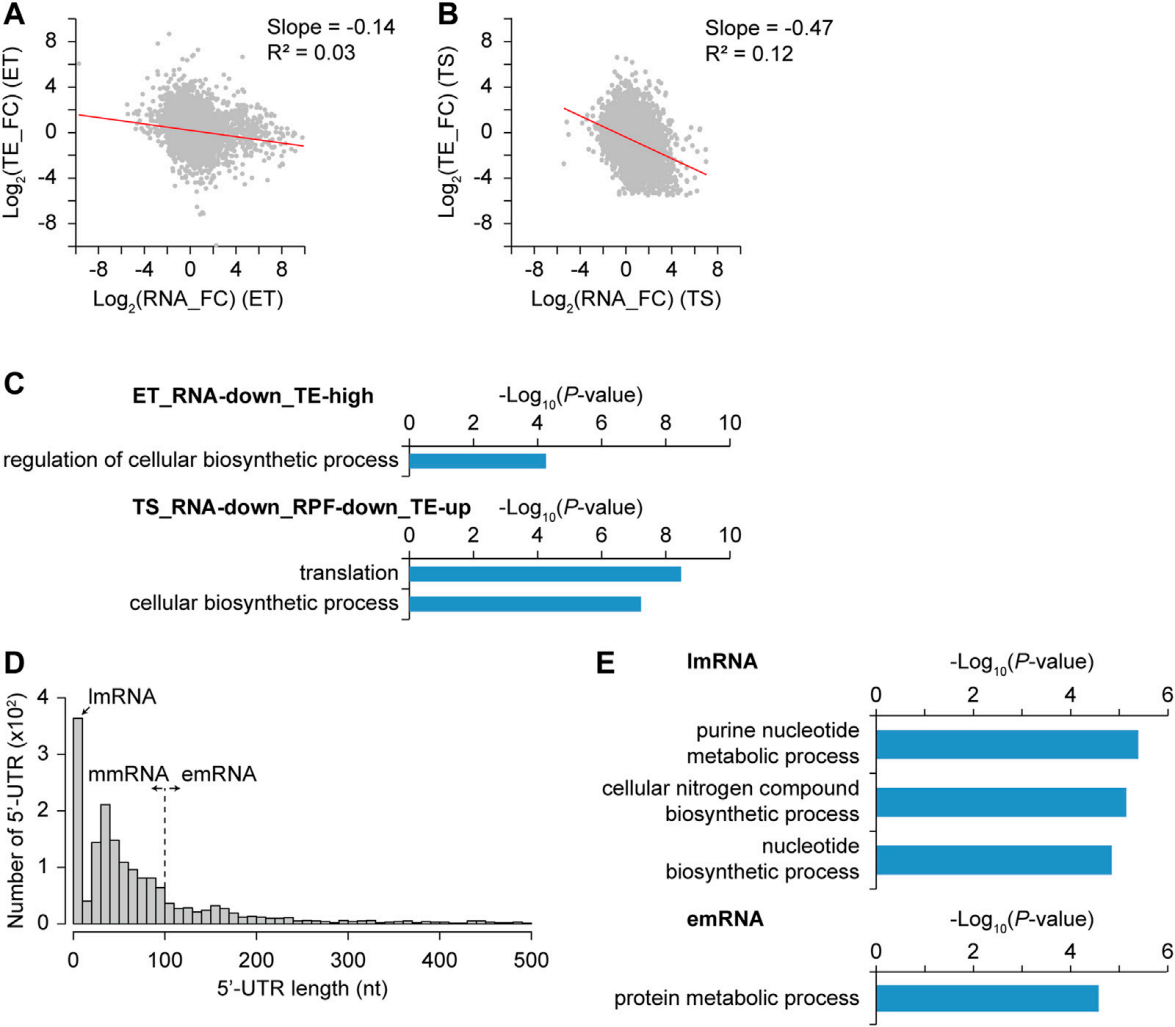
Differential transcription efficiency (TE) and its correlation with gene functions and 5′-UTR length. (A–B) Correlation between log_2_ RNA fold change and log_2_ TE fold change from **(A)** E to T phase and **(B)** T to S phase. Trend line (red) with their slope and R-squared value was indicated. **(C)** Different TE gene groups and their selected enriched gene functions of each group were represented with their −log_10_ *p*-value (GO-term enrichment, GO_Biological_Processes, hypergeometric test, Benjamini and Hochberg False Discovery Rate (FDR) correction (*q* < 0.05)). **(D)** 5′-UTR length distribution of genes with primary TSSs. They were classified based upon their 5′-UTR length, which are lmRNA (leaderless mRNA, 0–10 nt), mmRNA (moderate-leadered mRNA, 11–100 nt), and emRNA (extended-leadered mRNA, > 100 nt). **(E)** Enriched gene functions of lmRNA and emRNA group were represented with their −log_10_ *p*-value (GO-term enrichment, GO_Biological_Processes, hypergeometric test, Benjamini and Hochberg False Discovery Rate (FDR) correction (*q*-value < 0.05)). There were no enriched functions for mmRNA group.

In addition to the regulatory elements at the transcription level, those at the translation level could affect differential gene expression. The correlation between transcription and translation levels for individual genes was not high. Indeed, a discordance between mRNA and protein levels has been reported in many bacteria (De Sousa Abreu et al., 2009; Pospisil et al., 2020). Recently, translation buffering was observed in *S. coelicolor* and *S. clavuligerus*; the increase in the translation level of the secondary metabolism genes from early to later growth phases was not sufficient compared to the increase in their transcription levels (Jeong et al., 2016; Hwang et al., 2019). To analyze the translational regulation affecting the translation level change according to the transcription level change, the fold change of translation efficiency (TE) was calculated as the change in the ratio of RPF to RNA, representing the translating mRNA level among the total mRNA level. RNA and TE fold changes were negatively correlated with the slope of -0.14 and -0.47 in ET and TS phase transitions (Figures 6A,B). This trend of decreased TE in the TS phase transition was also abundant for streptomycin biosynthesis pathway genes and A-factor related genes (Supplementary Figure S4, S5). However, unlike the transcriptional DEG groups, functional enrichment analysis of genes based on the different RNA and TE patterns showed that only a few groups were enriched with the criteria of -log_10_(*p*-value) > 4 (Figure 6C). For example, genes with high TE (log_2_ (TE_FC) > 1) and RNA decrease (log_2_ (RNA_FC) < 1) during the ET phase transition (ET_RNA-down_TE-high group) have functional enrichment in the regulation of cellular biosynthetic processes. These regulator genes showed translational induction specifically at the T phase, which was previously observed for four clustered-situated regulators of representative antibiotics in *S. coelicolor* (Jeong et al., 2016). This result may be consistent with the enrichment of regulator genes in group IX (N-N) in Figure 2, implying specific translational regulation rather than transcriptional regulation. In another example, genes with TE increase [log_2_ (TE_FC) > 0] with both RNA and RPF decrease [log_2_ (RNA_FC) < 1 and log_2_ (RPF_FC) < 1] in the TS phase transition (TS_RNA-down_RPF-down_TE-up group) have functional enrichment in translation and cellular biosynthetic processes. This may be because these functions should be tightly regulated during translation to maintain a certain level, even if their transcription was less. Many ribosomal protein genes are involved in this group and are regulated through translational feedback by r-proteins (Nomura et al., 1984).

We speculated that differential TE changes for different genes in different growth phases would be affected by the regulation of translation initiation levels. Thus, the 5′-UTR length distribution, which contains translation initiation regulatory elements, including the ribosome binding site, was investigated (Figure 6D). According to the 5′-UTR length, mRNAs were divided into three groups: leaderless mRNA for 0–10 nt (lmRNA, n = 364), moderate-leadered mRNA for 11–100 nt (mmRNA, n = 1,010), and extended-leadered mRNA for 101–500 nt (emRNA, n = 338) to investigate their difference in TE. The distribution of the average TE fold change (TE_FC) in the three growth phases showed no significant differences among them (Supplementary Figure S9). Corroborating previous findings, lmRNAs may be differentially expressed in the stress conditions rather than in different growth phases (Cortes et al., 2013; Sawyer et al., 2020). Indeed, lmRNAs were functionally enriched in purine nucleotide metabolic processes and cellular nitrogen compound biosynthetic processes that may be related to stress responses (Figure 6E). In addition, emRNAs are functionally enriched in protein metabolic processes that are mostly ribosomal proteins and chaperones, which is consistent with the abundance of translational regulation on them (Nomura et al., 1984). Therefore, there was no significant correlation between 5′-UTR length and TE, but gene functions were differentially enriched according to 5′-UTR length, reflecting their common translation regulations.

### Regulatory Elements on Translation Elongation Level Affecting Differential Translation Efficiency

**FIGURE 7:**
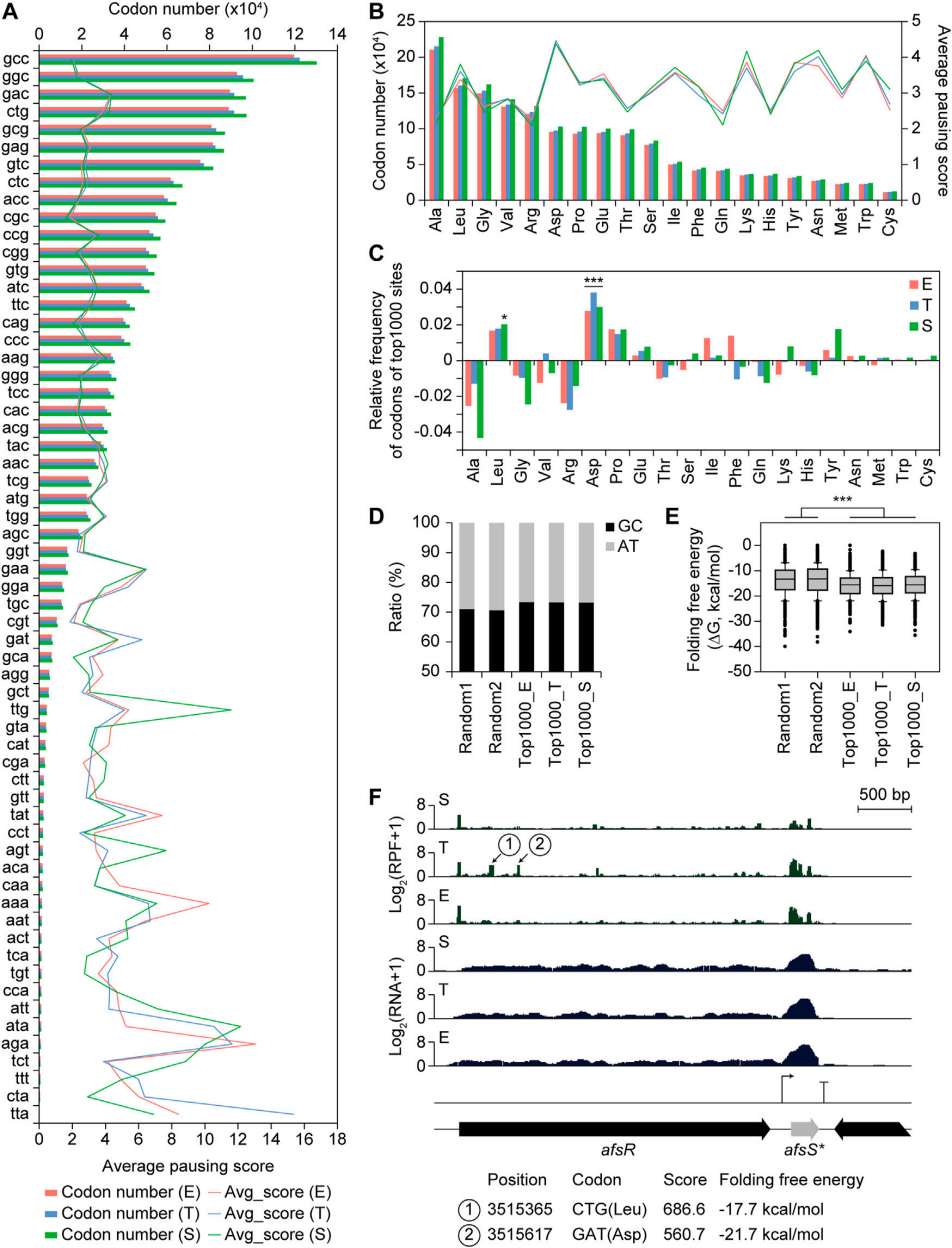
Codon usage and folding free energy of RNA structure affecting translation efficiency. **(A)** Average pausing score for 61 codons in A site of the ribosome, and their number of each codon. **(B)** Average pausing score for 20 amino acids in A site of the ribosome, and their number of codons corresponding to each amino acid. **(C)** Relative frequency of codons corresponding to each amino acid for top 1,000 pausing score sites compared to total sites. Statistical significance of the ratio was calculated by chi-square test. (***p < 0.01, and *p < 0.05). **(D)** GC ratio of the 40 nt downstream sequence from 3′-end boundary of ribosome at the pausing site of the two random position groups within CDS (except ten codons from both ends) and top 1,000 pausing score sites at three different growth phases. **(E)** Folding free energy of RNA structure of the 40 nt downstream sequence from 3′-end boundary of ribosome at the pausing site of the different groups in **(D)**. Statistical significance was calculated by Wilcoxon ranksum test. (***p < 0.01). **(F)** A profile example of potential ribosome pausing sites. Position, codon, pausing score, and folding free energy of RNA structure for the pausing sites were indicated.

Manual investigation of RPF profiles of the regulator genes in the ET_RNA-down_TE-high group (Figure 6C) revealed several RPF accumulation sites only in the T phase, suggesting differential TE by the regulation of the translation elongation level. These sites may be ribosome-pausing sites due to different codon usage. To designate the ribosome paused position at single-nucleotide resolution, the 3′-end position of the RPF reads was mapped. The middle position of the ribosome P site is obtained by shifting 14 nt upstream from the 3′-end position of RPF reads (Mohammad et al., 2019). Indeed, the normalized density of the 14 nt shifted 3′-end position count of RPF reads was enriched at the first position of start codons where ribosome binding was enriched and translation was initiated (Supplementary Figure S10A) (Giess et al., 2017). Thus, the pausing score of each position was calculated by the ratio of the 14 nt shifted 3′-end position count of RPF reads at the position to the average count of the involved CDS. The average pausing score of ribosome A-site codons showed an increasing trend as the total codon number was small, suggesting higher ribosome pausing in rare codons because of limited cognate aminoacyl-tRNAs (Figure 7A) (Nakahigashi et al., 2014). In particular, the TTA leucine rare codon showed the lowest codon number and the highest pausing score in the T phase among the 61 codons, rather than in the S phase. The TTA codons with the top three high pausing scores in the T phase were located within NADH dehydrogenase, beta-ketoacyl-ACP synthase III in HPQ melanin BGC, and aminoglycoside phosphotransferase family protein in germacradienol/geosmin BGC. Other AT-rich codons, including ATA, AGA, AAA, AAT, TAT, AGT, and GAA, showed relatively high average pausing scores in all or some growth phases and a relatively small codon number compared to other codons. Another leucine codon, TTG, showed a high average pausing score, particularly in the S phase, including TTG codons with the top three high pausing scores within the NRPS, NRPS-PKS hybrid, and PAS domain-containing genes in smBGCs. Therefore, TTA and TTG rare leucine codons seem to affect differential RPF accumulation in the T and S phases, respectively. The average pausing score according to the amino acid of the A-site codon showed no difference in the three growth phases, but was high in the order of Asp, Lys, Trp, Asn, Tyr, and Leu (Figure 7B).

Further, the relative frequency of codons and amino acids was investigated for the top 1,000 pausing score sites compared to the total number of sites in each growth phase. The GAC, CTG, and CCG codons showed the highest frequency for the top 1,000 pausing sites encoding Asp, Leu, and Pro, respectively (Supplementary Figure S10B). Indeed, Asp, Leu, and Pro showed the highest frequency for the top 1,000 pausing sites, however, Asp, Leu were significantly high in all three phases and in the S phase, respectively (Figure 7C). The regulator genes in ET_RNA-down_TE-high group (Figure 6C) also showed high pausing scores for the Asp and Leu amino acids in the T phase. However, the relative frequency distribution of amino acids of the top 1,000 pausing sites within PKS and NRPS biosynthetic genes showed different trends in the top 1,000 pausing sites, suggesting regulations specific for individual sites (Supplementary Figure S10C). The mechanisms of high pausing in specific amino acid sites only in specific genes during a certain growth phase remain to be elucidated.

Ribosome pausing could also occur by mRNA stem-loop structures inhibiting translocation or hindering A-site tRNA binding (Bao et al., 2020). To investigate the correlation between the mRNA structure and pausing score, the GC ratio and folding free energy of +40 nt sequences from the 3′-end position of RPF reads were calculated for the top 1,000 pausing score sites in each growth phase. Compared to the two independent groups of random CDS positions, except 100 nt from both ends, the GC ratio was higher (71.1 and 70.7% for the random group, and 73.5, 73.4, and 73.2% for the top 1000 E, T, and S groups, respectively), and the folding free energy was significantly lower for all growth phases (Figures 7D,E). This result suggests that the RNA structure downstream of the ribosome affects ribosome pausing, but there was less correlation between individual sites within the top 1,000 pausing sites, suggesting many additional specific regulations, including codon usage. In addition, specific sequence motifs for RNA-binding proteins or RNA-degrading enzymes may be present; however, they were not found in this study.

One example of a pausing site was *afsR* (Figure 7F). There were two sites with accumulated RPF reads only in the T phase, namely, the CTG (Leu) and GAT (Asp) codons. The folding free energy of their downstream sequence were −17.7 and −21.7 kcal/mol, respectively, which were lower than the median value of those of the top 1,000 pausing sites (-15.5 kcal/mol). Thus, both codon usage and RNA-folding free energy at these sites may affect the accumulation of RPF reads. AfsR is a SARP family regulator that is activated by the sensor kinase AfsK and other kinases (Horinouchi, 2003). Although the AfsK/AfsR system is involved in the response of aerial mycelium formation to glucose but not to streptomycin production in *S. griseus* (Umeyama et al., 1999), overexpression of AfsR activates the production of various secondary metabolites in other *Streptomyces* species, such as pikromycin in *S. venezuelae* ATCC 15439 (Maharjan et al., 2009), actinorhodin (ACT), and prodiginine (RED) in *S. coelicolor* and *S. lividans* (Horinouchi et al., 1989). Therefore, RPF accumulation sites within *afsR* in the T phase may be the translational regulatory element of *afsR*, related to the activation of smBGCs. On the other hand, an intergenic TU was observed in the downstream region of *afsR*, showing high RNA and RPF profiles. This TU contained a small ORF, the AfsS homolog ORF4 (Umeyama et al., 1999; Santos-Beneit et al., 2011). AfsS activates the transcription of ACT and RED smBGC genes in *S. coelicolor*, although its mechanism is still unclear (Kim et al., 2018). Although *afsS* is non-DEG, it is a potential engineering target of smBGC activation found in intergenic TU. In summary, codon usage and RNA structure downstream of ribosome-bound sites affected differential TE at the translational elongation level.

## Discussion

**FIGURE 8:**
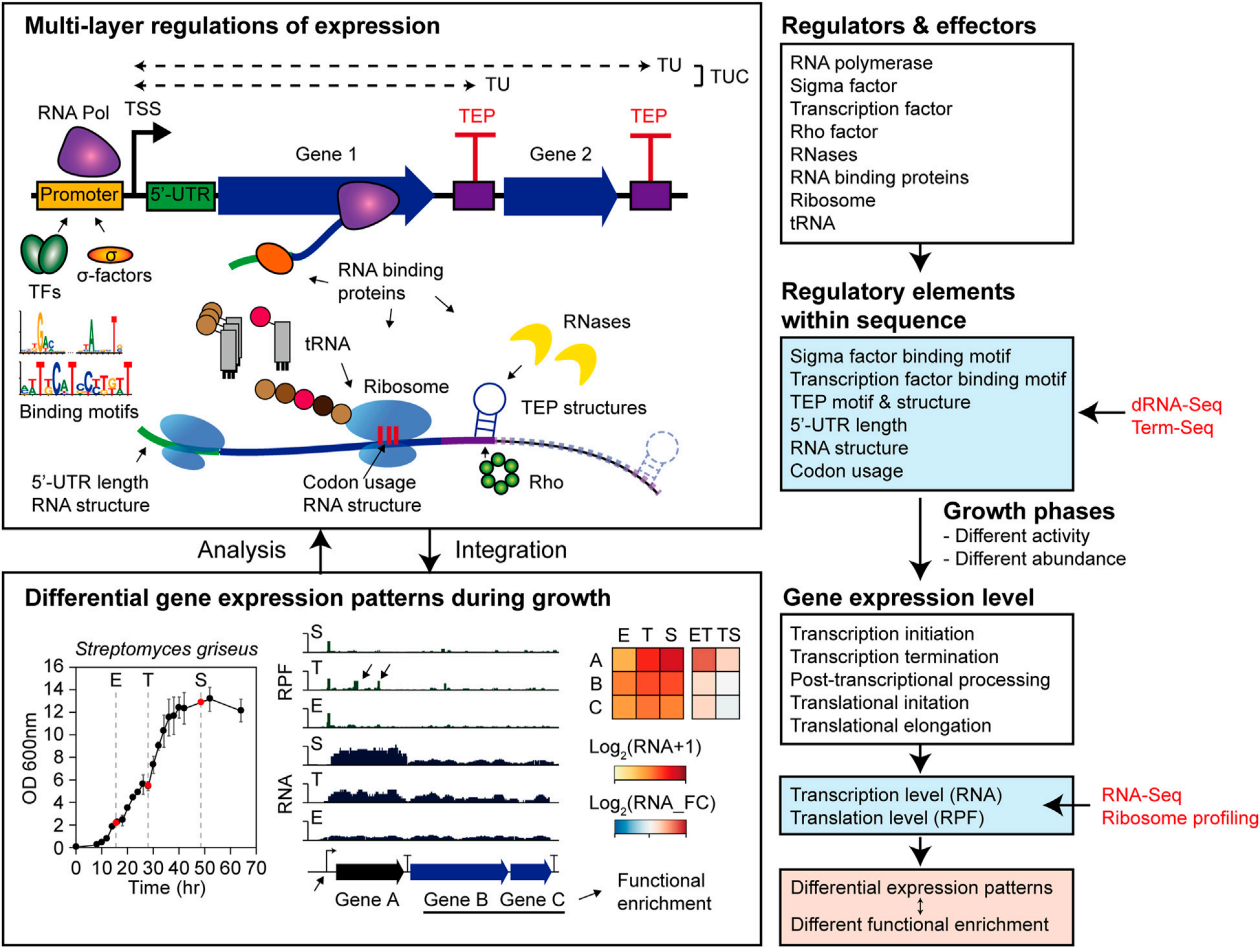
Overall conclusion scheme of this study.

This study systematically analyzed the regulatory elements of transcription and translation levels affecting DEGs in *Streptomyces griseus* NBRC 13350 during their growth and functional enrichments (Figure 8). Gene functions were enriched according to the transcription patterns expected to be integrated to orchestrate dynamic cellular status, resulting in a metabolic shift. In the ET phase transition, cellular growth functions, including carbon source consumption, cell division by cell wall component recycling, and energy production, were upregulated. Moreover, the functions of translation, aromatic compound catabolism, and nitrogen utilization were downregulated, which may be negatively related to rapid growth and development. In the TS phase transition, a general decreasing trend in cell growth and energy production and an increasing trend in secondary metabolism were observed.

In a previous study, the metabolic shift by the cascade from A-factor was predicted to occur rapidly, and the addition of the critical concentration of A-factor at the 12 h growth phase in YMPD medium activated the AdpA regulon genes within 30 min (Hara et al., 2009). However, in this study, the high transcription level of *adpA* in the E phase (15.5 h) suggests that this A-factor cascade had already started before the E phase (Supplementary Figure S5). In addition, rapidly activated genes after A-factor addition in the previous study were mostly non-differentially expressed and even downregulated in the ET phase transition in this study, supporting the initiation of the A-factor cascade at an earlier time point than the E phase. Also, upregulation of *afsA* transcription in the later growth phase was observed in this study, which was also reported in a previous study (Kato et al., 2007), but the underlying mechanism remains unknown. Therefore, further systematic data at additional time points and in various media should be accumulated to attain a deeper understanding of this complex metabolic shift.

Next, to elucidate the regulatory elements in the promoter affecting differential transcription patterns at the transcription initiation level, the binding motifs of sigma factors and transcription factors were searched. As a result, the conserved binding motifs of sigma and transcription factors differed in each differential transcription pattern group. In addition, the binding motifs and regulons of one potential sigma factor and four potential TFs were elucidated. Further, with RNA-seq and dRNA-seq under various conditions, potential binding motifs of other sigma factors and transcription factors could be efficiently predicted. Additional ChIP-Seq and *in vitro* binding assays would validate the predicted regulons to understand the complex transcriptional regulatory network.

By determining genome-wide TEPs by Term-seq, the potential regulatory elements of transcription termination and post-transcriptional processing at the 3′-end of the transcripts were investigated. Motif TEPs show features of intrinsic terminators, while Structured non-motif TEPs are likely I-shaped intrinsic terminators of the GC-rich eubacterial genome (Mitra et al., 2009), or enriched 3′-end termini of Rho-dependent-terminated transcripts (Dar and Sorek, 2018). Unstructured non-motif TEPs may be Rho-dependent termination sites with diffuse features or unknown post-transcriptional processing sites (Jin et al., 1992). These unique features of TEPs were analogous to those of *S. lividans* (Lee et al., 2019) and *S. clavuligerus* (Hwang et al., 2021).

In addition, TUs and TUCs were determined from TSSs and TEPs to provide genome-wide information on the TU isoforms during growth. Moreover, transcript abundance within TUC was differentially changed in non-terminal TEPs according to the features of the non-terminal TEP. For example, the squalene cyclase *hopE* gene and its downstream modification genes showed differential transcription patterns in the ET phase transition that were considered to be regulated by non-terminal TEP. The biological functions of hopanoids include the control of cell membrane fluidity and integrity; however, many structural variants of hopanoids have also been reported to have other functions, including low pH tolerance, antimicrobial resistance, alignment of extracellular cellulose microfibrils, and plant–bacteria interactions (Kannenberg and Poralla, 1999; Poralla et al., 2000; Schmerk et al., 2011; Belin et al., 2018). Further mechanistic studies of this regulatory element of non-terminal TEP would expand our understanding of hopanoid function and regulation. Moreover, premature TUs were detected that potentially affect differential transcription patterns of downstream genes, such as the cobalamin riboswitch of the methylmalonate-semialdehyde dehydrogenase gene, and its downstream genes involved in valine degradation to propanoyl-CoA (Zhang et al., 1996). Although cobalamin is an essential cofactor for several enzymes with broad functions (Borovok et al., 2006; Takano et al., 2015), no cobalamin-related studies have been conducted on valine degradation enzymes. Since valine and propanoyl-CoA are both related to peptide or polyketide-type secondary metabolites as precursors, further studies of cobalamin riboswitch regulation of these genes could expand the understanding of secondary metabolism.

The translation level regulation in genes with differential transcript patterns was analyzed in terms of the TE fold change during growth. For translation initiation, 5′-UTR length did not show a significant correlation with the TE fold change during growth. However, specific functional enrichments of lmRNA and emRNA suggested that lmRNAs may play important roles in other conditions, such as stress conditions, rather than different growth phases (Cortes et al., 2013). Translation pausing was more likely to occur in the AT-rich rare codon and in some specific amino acids, including Asp, Leu, and Pro, depending on the growth phase. The significantly high pausing score at aspartic acid may be related to intracellular nitrogen metabolism connected to glutamate or smBGCs producing peptide-type metabolites. Moreover, abundant RPF accumulation at specific regulatory gene sites was observed in the T phase. This RPF accumulation site may be the translation pausing site due to the lack of rare codon tRNA or cognate amino acid, which could be the engineering point for enhancing translation. Otherwise, this site may be related to ribosome spacing and RNase degradation as in the case of rare codon encoded in *E. coli rpoS* gene, which is the regulatory element playing a positive role in its expression (Kolmsee and Hengge, 2011). In addition, the higher folding free energy of the RNA structure downstream of the ribosome seemed to inhibit translocation or hindering A-site tRNA binding, resulting in ribosome pausing.

In conclusion, the metabolic shift during *S. griseus* growth was investigated in terms of transcriptome and translatome. A pipeline for systematic analysis of differentially expressed genes during growth was provided. The potential regulatory elements found in this study may facilitate this rational engineering design for enhancing secondary metabolite production.

## Data Availability

RNA-seq, dRNA-seq, and Term-seq data were deposited in the European Nucleotide Archive (ENA) under the accession number PRJEB40918. Ribosome profiling data were deposited in the European Nucleotide Archive (ENA) under accession number PRJNA575265.
