# Supplementary material for: System-Level Analysis of Transcriptional and Translational Regulatory Elements in Streptomyces griseus
Source: Front Bioeng Biotechnol. 2022 Feb 25;10:844200. doi: 10.3389/fbioe.2022.844200 (PMC8914203; doi:10.3389/fbioe.2022.844200)
Supplement: Supplementary file 3 [file DataSheet1.PDF]

Supplementary Material

1.1 Supplementary Figures

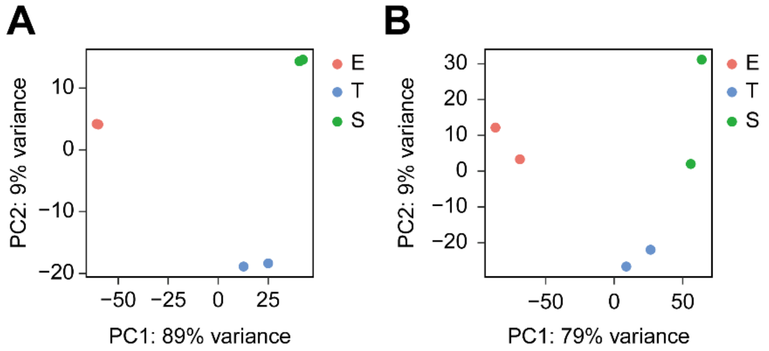

Supplementary Figure 1. Quality assessment of RNA-Seq and ribosome profiling data. (A) PCA reproducibility plot of RNA-Seq. **(B)** PCA reproducibility plot of ribosome profiling.

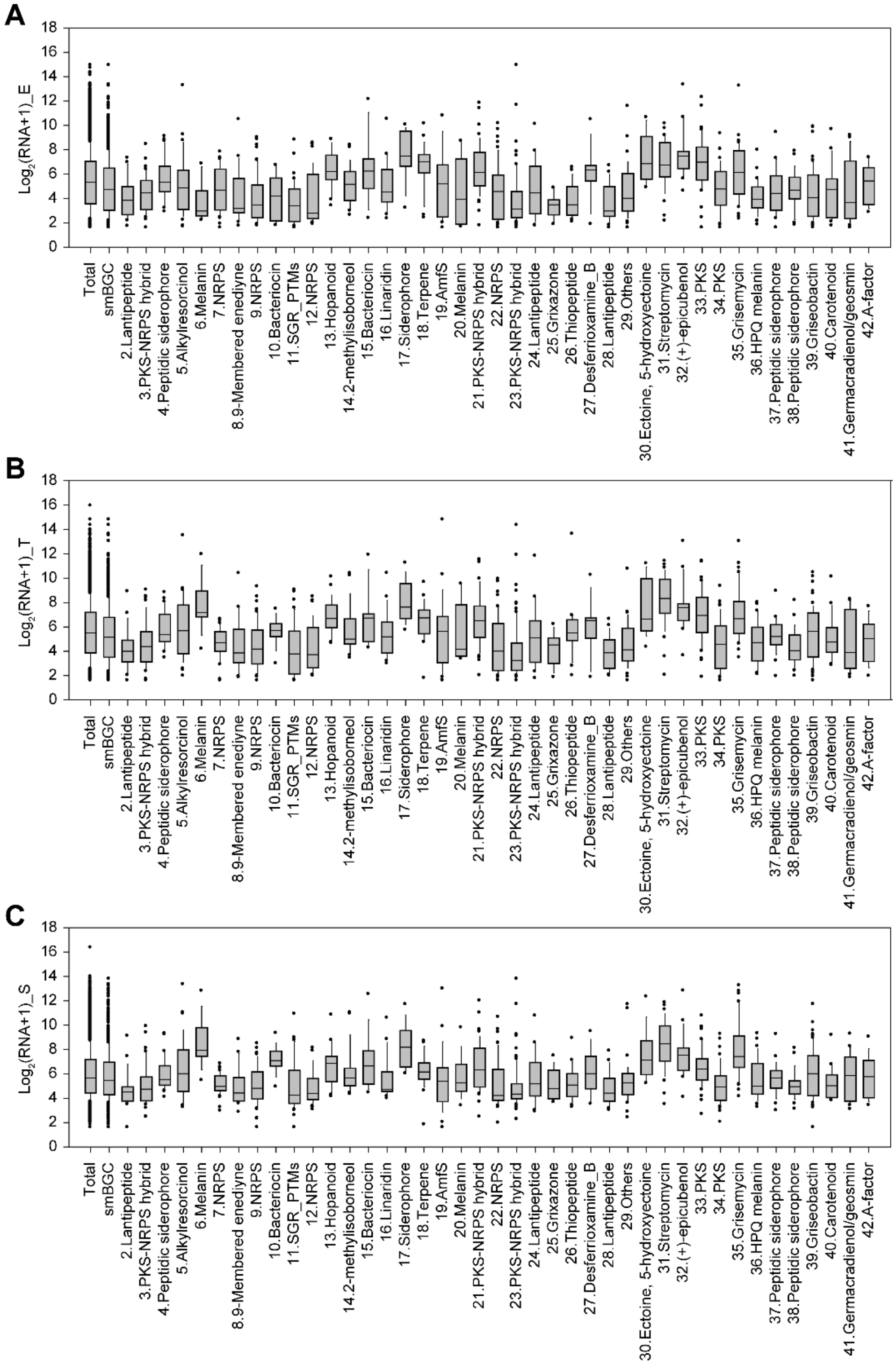

Supplementary Figure 2. Normalized transcription level distribution of secondary metabolite biosynthetic gene clusters (smBGCs) at three growth phases. Each smBGC was represented as the number, that is the order of their genomic location, and the name of validated or predicted product from antiSMASH or previous reports. In the absence of a predicted product, the name of the production enzyme was instead stated.

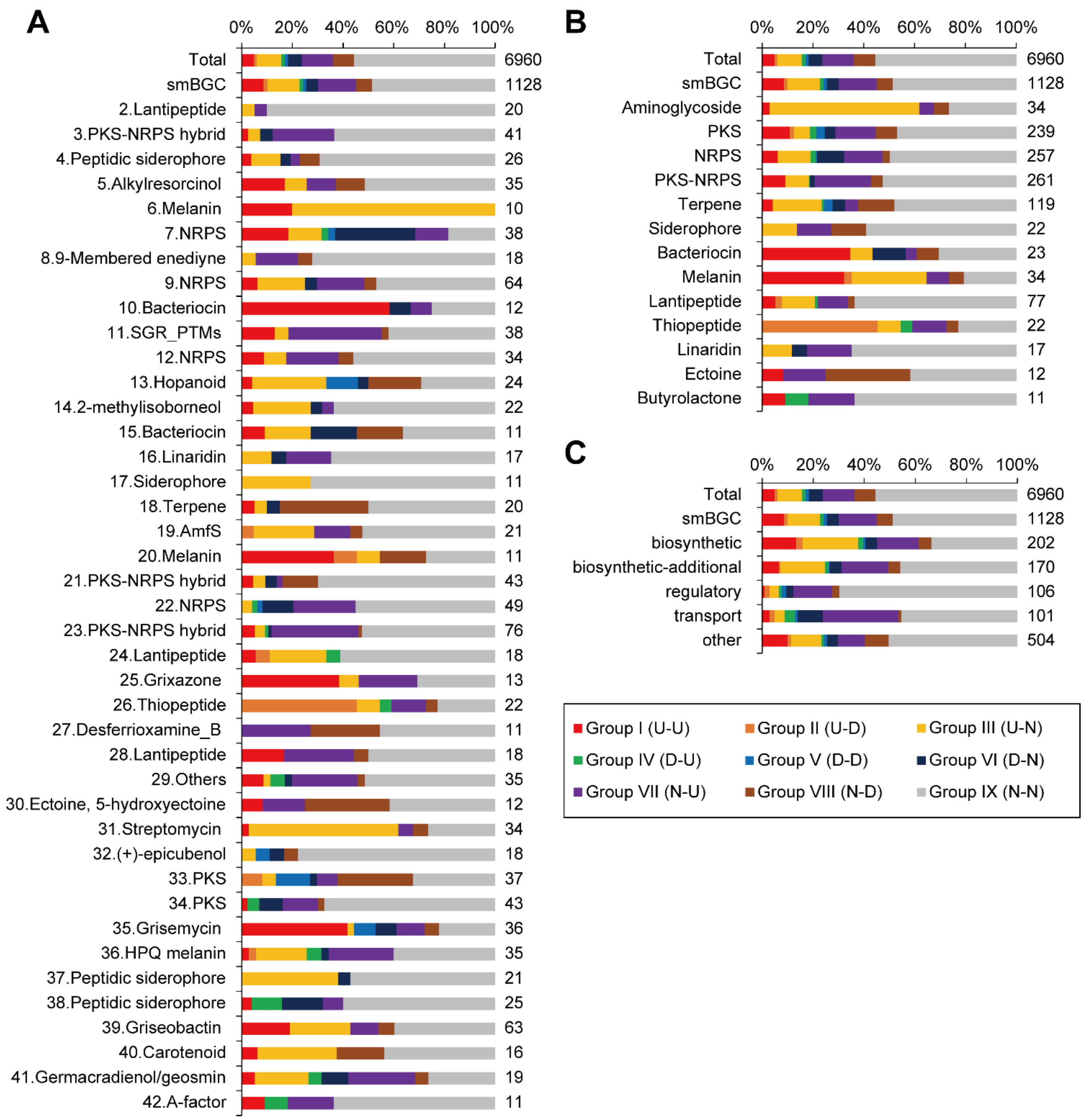

Supplementary Figure 3. Number frequency distribution of the nine gene expression pattern groups in Fig 2 for (A) smBGCs, (B) smBGC types, and (C) smBGC gene functional categories from antiSMASH. Total gene numbers of each group were indicated at the right of the bar graph.

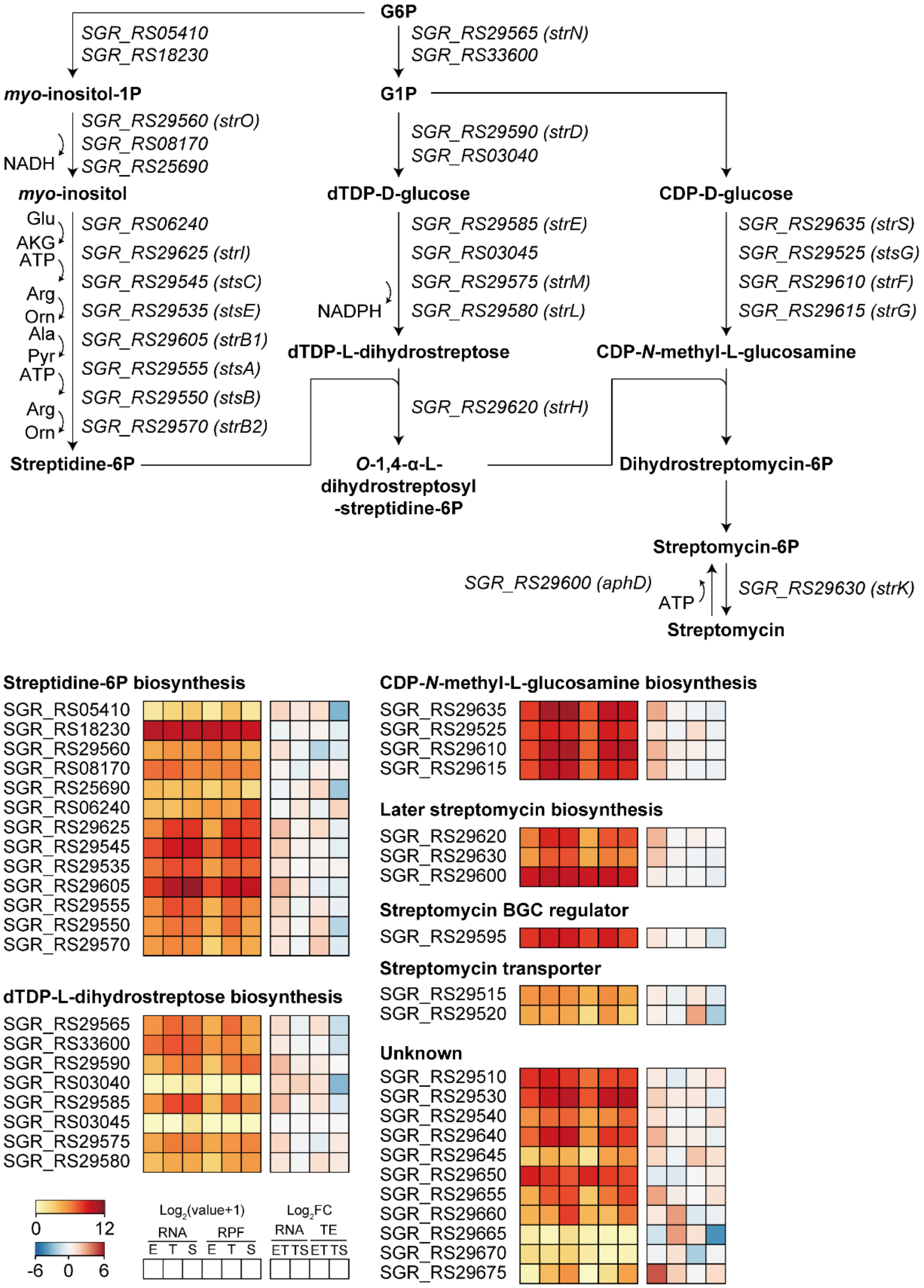

Supplementary Figure 4. Gene expression pattern of streptomycin biosynthetic pathway. Overall biosynthetic pathway of streptomycin and their governing genes were indicated. Transcription level, translation level, transcription fold change, and translation efficiency fold change of genes were indicated.

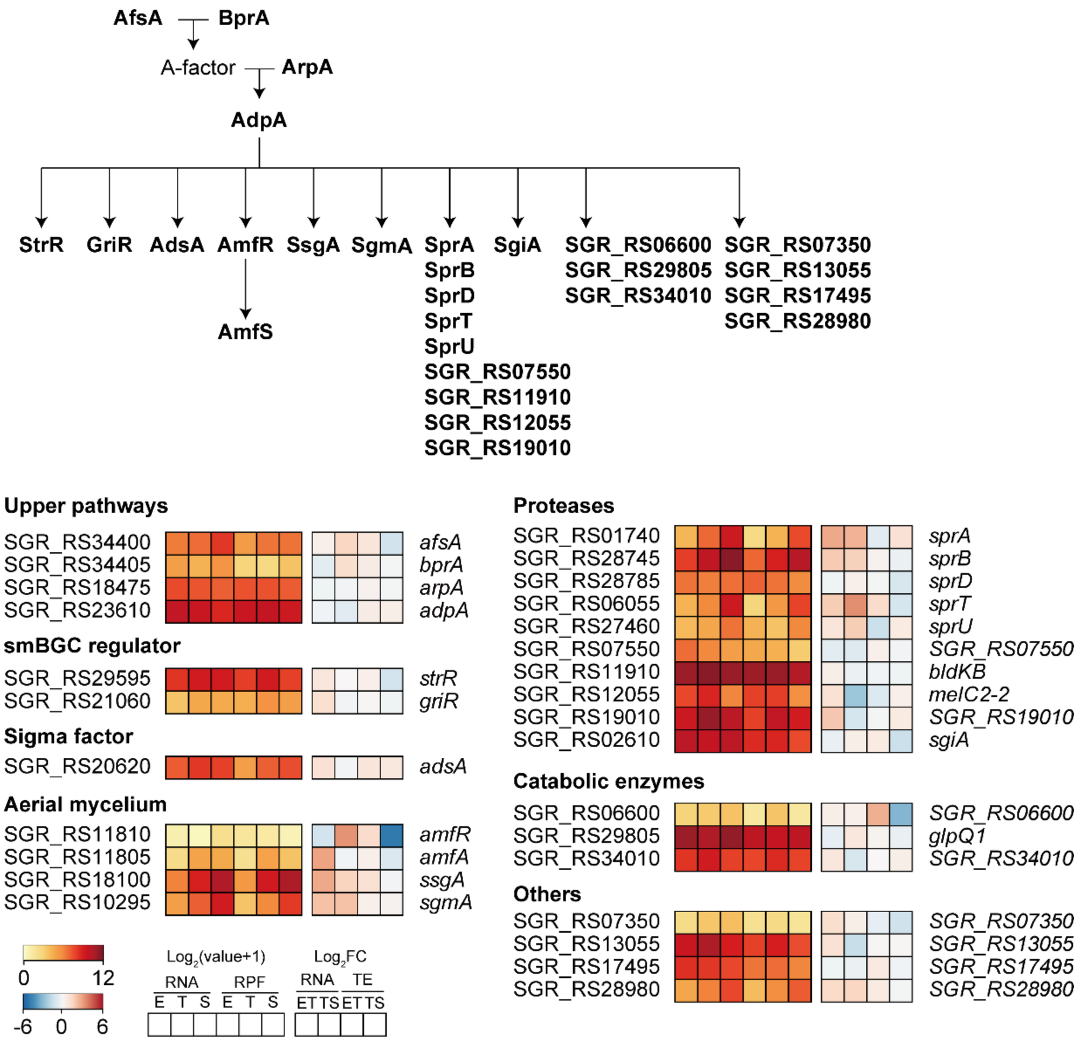

Supplementary Figure 5. Gene expression pattern of A-factor related transcription regulatory network. Overall network including transcription factors and representative regulons reported from previous studies were indicated. Transcription level, translation level, transcription fold change, and translation efficiency fold change of genes were indicated.

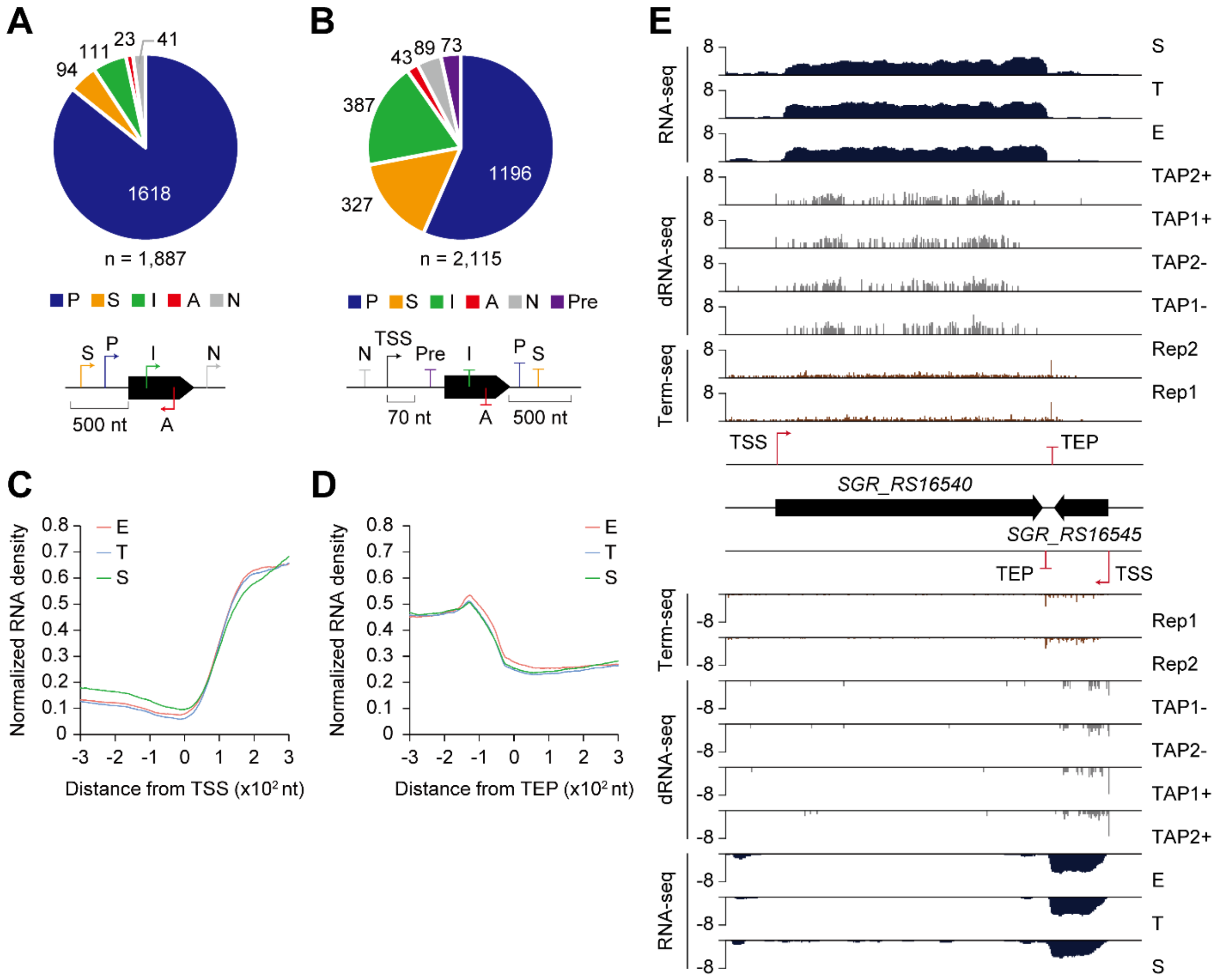

Supplementary Figure 6. (A) Classification of determined TSSs according to their relative genomic position from the gene. A total of 1,618 TSSs were classified as primary TSSs (P), which have the highest peak intensity among the peaks located in the 500 nt upstream region of the associated gene, and 94 TSSs were assigned as secondary TSSs (S), which were located at the same upstream region of primary TSSs. Among the remaining TSSs, 111 internal TSSs (I) located inside of ORFs and 23 antisense TSSs (A) located inside of the opposite strand of ORFs were determined. Lastly, the remaining 41 TSSs were classified as intergenic TSSs (N), which were located in the intergenic region excluding 500 nt upstream regions of the associated genes. **(B)** Classification of determined TEPs according to their relative genomic position from the gene. A total of 1,196 TEPs were categorized as primary TEPs (P), which showed the highest peak intensity among the TEPs located at the 500 nt downstream region of the associated gene, and 327 TEPs in the same region except primary TEPs were categorized as secondary TEPs (S). Among the remaining TEPs, 387 TEPs located within the ORF of the sense strand were categorized as internal TEPs, and 73 TEPs were categorized as premature TEPs (Pre) which were located at the region between 70 nt downstream of the primary TSS and the first position of the start codon of the associated gene. Also, 43 TEPs located within the ORF of the opposite strand were categorized as antisense TEPs (A), and the remaining 89 TEPs were categorized as intergenic TEPs (N). **(C)** Normalized RNA read count density at –300 to +300 nt window of TSSs. **(D)** Normalized RNA read count density at –300 to +300 nt window of TEPs. **(E)** An example of RNA-seq, dRNA-seq, and Term-seq profiles.

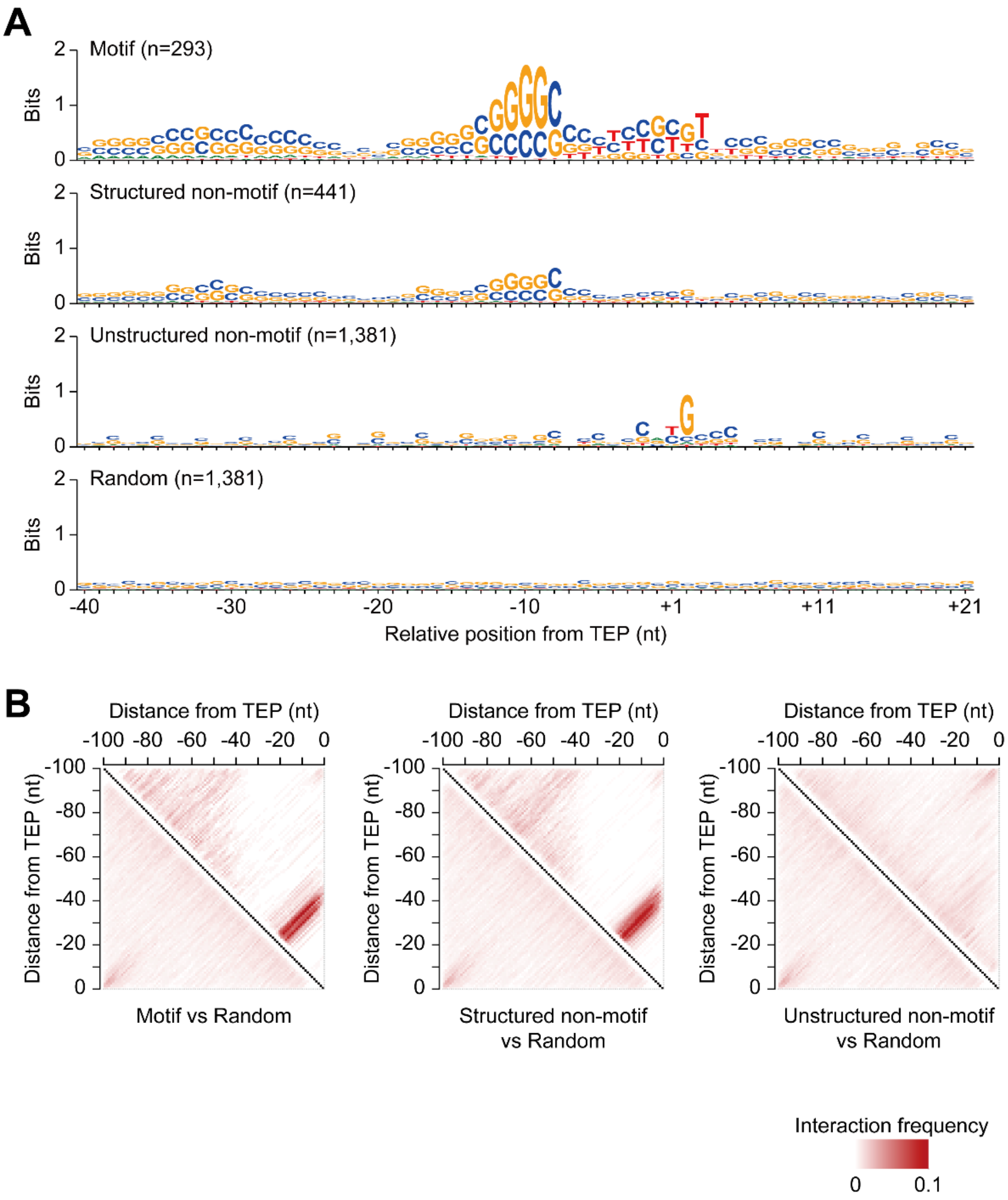

Supplementary Figure 7. Characteristics of three subcategories of TEPs. (A) Sequence alignments of –40 to +21 nt region from three subcategories TEPs and random intergenic positions. **(B)** Interaction frequency between two nucleotides located at 100 nt upstream region of TEPs. Motif (left graph, upper triangle), structured non-motif (middle, upper triangle), and unstructured non-motif (right graph, upper triangle) TEP subcategories were compared to random positions (lower triangle of all three graphs).

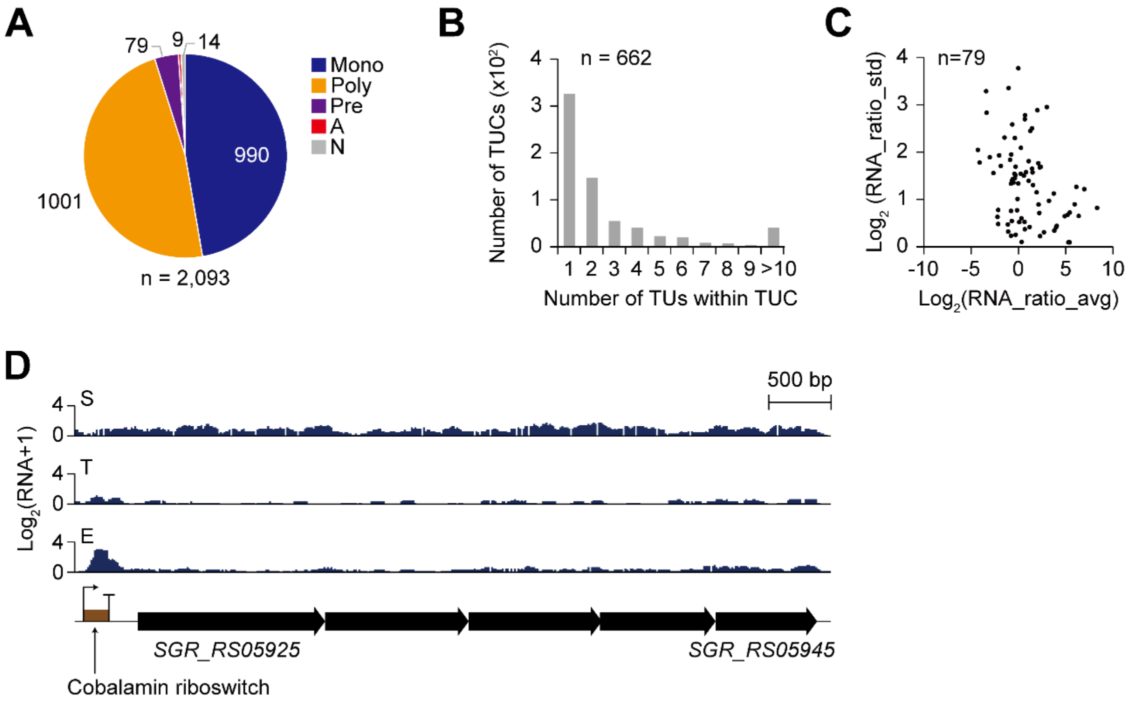

Supplementary Figure 8. Characteristics of transcription unit clusters (TUCs) and identification of premature transcription units affecting differential transcription levels of downstream genes. (A) Classification of transcription units (TUs) based on the number of genes within each TU and their location relative to the genes. **(B)** The number distribution of TUs within each TUC. **(C)** Distribution of the average and standard deviation of $\log_2$ RNA read count ratio of the premature TU to the CDS at the three growth phases. **(D)** An example of a premature TU affecting differential transcription level of its downstream genes at different growth phases. Cobalamin riboswitch in the premature TU was predicted from Rfam database.

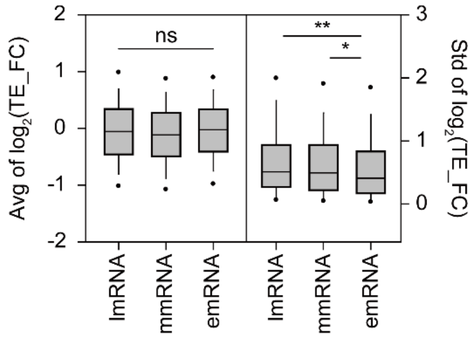

Supplementary Figure 9. Potential effects on translation efficiency at translation initiation level. Distribution of average (left) and standard deviation (right) of $\log_2$ TE fold change of different 5′-UTR length gene groups in **Figure 6D**. 5% outliers were excluded. Statistical significances between nonTSS group and other three groups, and between emRNA group and lmRNA or mmRNA group were calculated by Wilcoxon ranksum test. (***$P < 0.001$, **$P < 0.01$, and *$P < 0.05$).

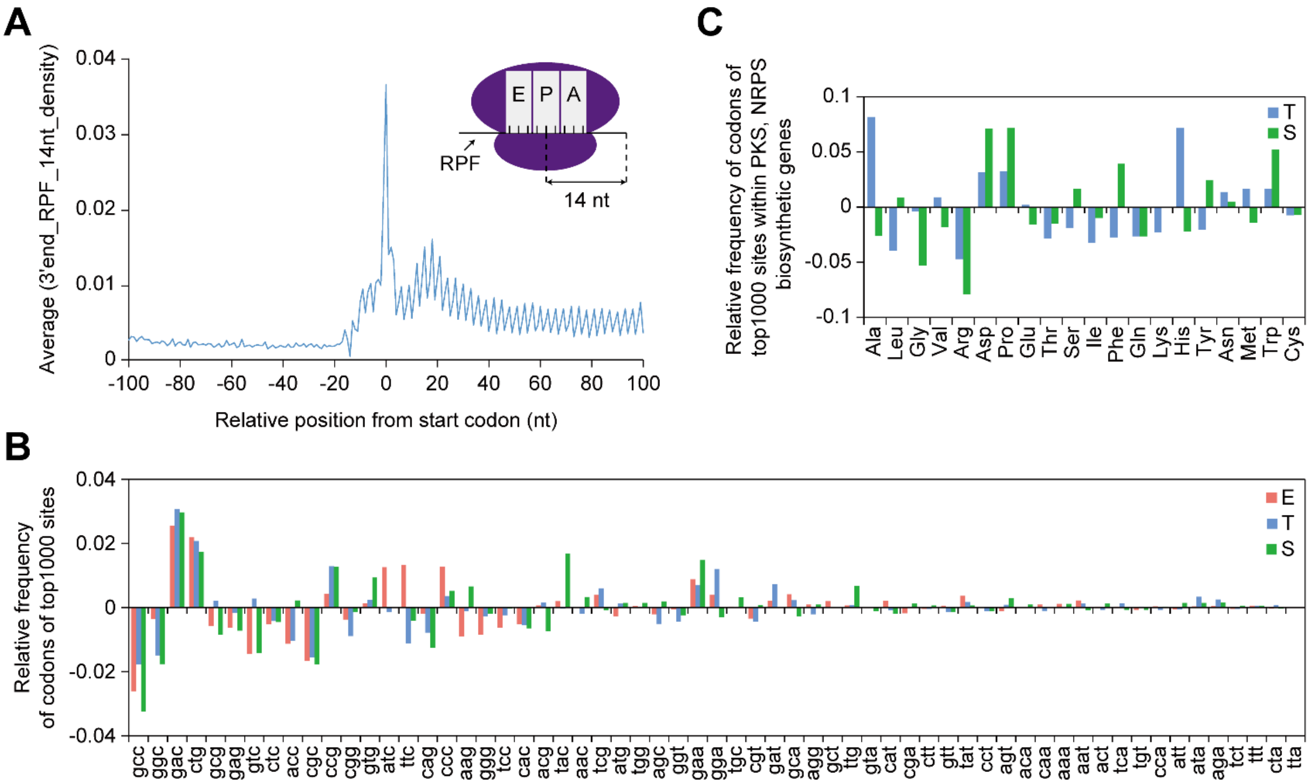

Supplementary Figure 10. Potential effects on translation efficiency at translation elongation level. (A) Average normalized density of the count of 3′-end of ribosome profiling reads which are shifted by 14 nt upstream to the position. **(B)** Relative frequency of each codon for top 1,000 pausing score sites compared to total sites at three different growth phases. **(C)** Relative frequency of codons corresponding to each amino acid for top 1,000 pausing score sites within PKS and NRPS biosynthetic genes compared to total sites at two different growth phases.

Supplementary Table 1. Transcription and translation levels and fold changes during growth in *S. griseus* NBRC 13350. Expression values were normalized to TPM. 'FC' and 'pval' means $\log_2$ (fold change) value and *P*-value from DESeq2. Null value or no description were represented by '.' or 'NA'. **(xlsx)**

Supplementary Table 2. smBGC list for *S. griseus*. Cluster name was determined by the integration of (1) the number of genomic positions and (2) cluster product or cluster type. Cluster types and products were predicted using antiSMASH or previous reports. **(xlsx)**

Supplementary Table 3. Information on all transcription start sites (TSSs), transcript 3′ end sites (TEPs), transcription units (TUs), and transcription unit clusters (TUCs). The abundance of TSS and TEP was the normalized 5′-end or 3′-end count of the mapped reads. 'No.' denotes the number of content items. **(xlsx)**
